# Stabilizing heterochromatin by DGCR8 alleviates senescence and osteoarthritis

**DOI:** 10.1038/s41467-019-10831-8

**Published:** 2019-07-26

**Authors:** Liping Deng, Ruotong Ren, Zunpeng Liu, Moshi Song, Jingyi Li, Zeming Wu, Xiaoqing Ren, Lina Fu, Wei Li, Weiqi Zhang, Pedro Guillen, Juan Carlos Izpisua Belmonte, Piu Chan, Jing Qu, Guang-Hui Liu

**Affiliations:** 1 National Laboratory of Biomacromolecules, CAS Center for Excellence in Biomacromolecules, Institute of Biophysics, Chinese Academy of Sciences, 100101 Beijing, China; 2 State Key Laboratory of Stem Cell and Reproductive Biology, Institute of Zoology, Chinese Academy of Sciences, 100101 Beijing, China; 3 State Key Laboratory of Membrane Biology, Institute of Zoology, Chinese Academy of Sciences, 100101 Beijing, China; 4 University of Chinese Academy of Sciences, 100049 Beijing, China; 5 Institute for Stem Cell and Regeneration, Chinese Academy of Sciences, 100101 Beijing, China; 6 Advanced Innovation Center for Human Brain Protection, National Clinical Research Center for Geriatric Disorders, Xuanwu Hospital Capital Medical University, 100053 Beijing, China; 7 Key Laboratory of Genomics and Precision Medicine, Beijing Institute of Genomics, Chinese Academy of Sciences, 100101 Beijing, China; 8 Clinica Cemtro. Av. del Ventisquero de la Condesa, 42, 28035 Madrid, Spain; 9 Gene Expression Laboratory, Salk Institute for Biological Studies, La Jolla, CA 92037 USA; 10 Key Laboratory of Regenerative Medicine of Ministry of Education, Institute of Aging and Regenerative Medicine, Jinan University, 510632 Guangzhou, China; 11 Beijing Institute for Brain Disorders, Capital Medical University, 100069 Beijing, China

**Keywords:** Ageing, Regeneration, Senescence, Chromatin remodelling

## Abstract

DiGeorge syndrome critical region 8 (DGCR8) is a critical component of the canonical microprocessor complex for microRNA biogenesis. However, the non-canonical functions of DGCR8 have not been studied. Here, we demonstrate that DGCR8 plays an important role in maintaining heterochromatin organization and attenuating aging. An N-terminal-truncated version of DGCR8 (DR8^dex2^) accelerated senescence in human mesenchymal stem cells (hMSCs) independent of its microRNA-processing activity. Further studies revealed that DGCR8 maintained heterochromatin organization by interacting with the nuclear envelope protein Lamin B1, and heterochromatin-associated proteins, KAP1 and HP1γ. Overexpression of any of these proteins, including DGCR8, reversed premature senescent phenotypes in DR8^dex2^ hMSCs. Finally, DGCR8 was downregulated in pathologically and naturally aged hMSCs, whereas DGCR8 overexpression alleviated hMSC aging and mouse osteoarthritis. Taken together, these analyses uncovered a novel, microRNA processing-independent role in maintaining heterochromatin organization and attenuating senescence by DGCR8, thus representing a new therapeutic target for alleviating human aging-related disorders.

## Abstract (web summary)

DGCR8 is a component of the canonical microprocessor complex for microRNA biogenesis. Here the authors implicate DGCR8 in heterochromatin maintenance and aging attenuation independent of its miRNA-processing activity through Lamin B1, KAP1 and HP1 interaction. DGCR8 overexpression can alleviate aging and senescence

## Introduction

Aging is a crucial driving force in human degenerative disorders. Possible mechanisms for organismal aging include loss of heterochromatin^1^, free radicals, programmed senescence, telomere shortening, genomic instability^2^, nutritional intake, and growth signaling^3^. Stem cell aging is newly recognized as an important culprit in organismal aging^3,4^. For example, aging of mesenchymal stem cells (MSCs) has been shown to drive aging-associated tissue degeneration^5^. MSCs, which have the potential to differentiate into mesodermal lineages like osteoblasts, chondrocytes and adipocytes, can be isolated from various tissues including bone marrow, cord blood, adipose tissue and dental pulp^6^. Premature depletion of MSCs is observed in patients with Hutchinson-Gilford progeria syndrome (HGPS) and Werner syndrome (WS), two premature aging diseases that are associated with accelerated atherosclerosis, osteoporosis, and osteoarthritis^7,8^. Despite numerous studies showing that MSCs play pivotal roles in tissue rejuvenation, regeneration, and repair by differentiating into various somatic cell types^6^, little is known about the key regulators of MSC aging.

Aging-associated declines in stem cell functionality are often accompanied by epigenetic changes, such as changes in genomic DNA methylation, histone modifications, and chromatin remodeling enzymes^3,4,9,10^. Heterochromatin domains are structurally inaccessible and usually transcriptionally inactive. These domains are established during early stages of embryogenesis and are gradually lost with aging, resulting in the de-repression of normally silenced genes^1^. Heterochromatin is associated with specific proteins, such as heterochromatin protein 1 (HP1), and specific histone modifications, like H3K9me3 (trimethylated histone H3 at lysine-9)^11^, both of which decrease along with heterochromatin loss. In MSCs derived from patients with HGPS or WS, reduction of heterochromatin is observed, along with decreased levels of HP1, H3K9me3, and H3K27me3^4,7,9,12^. Whereas heterochromatin loss drives human MSC (hMSC) aging^9,13^, the re-establishment of heterochromatin alleviates premature aging and promotes longevity in *Drosophila* and human cells^14,15^, suggesting that the maintenance of heterochromatin organization could be an effective therapeutic intervention against aging.

DGCR8, which is known as Pasha in flies and worms, is a 773-amino-acid (a.a.) protein critical for microRNA (miRNA) biogenesis via the formation of a microprocessor complex with Drosha. This complex processes primary miRNA in the nucleus^16^. The C-terminal tail (a.a. 685-773) of DGCR8 binds to and stabilizes Drosha; the dsRNA-binding domain (a.a. 510-684) interacts with pri-miRNAs to increase binding affinity; the RNA-binding heme domain (a.a. 276-498) ensures processing accuracy and enhances efficiency. In contrast, little is known about the N-terminal region (a.a. 1-270), except that it contains a nuclear localization signal (NLS)^17,18^. During the past decades, studies on DGCR8 have primarily focused on its miRNA-processing function. DGCR8-knockout mouse embryonic stem cells (mESCs) have defects in proliferation and differentiation due to the global loss of miRNAs^19^. DGCR8 deletion in murine B-cell progenitors impairs the transition from pro- to early pre-B cell stage, recapitulating the phenotypes observed in miR17-92 cluster knockout mice^20^. However, it was recently reported that UV-induced phosphorylation of DGCR8 at S153 mediates transcription-coupled nucleotide excision repair of UV-induced DNA lesions, independent of its miRNA-processing activity^21^, suggesting that DGCR8 may have non-canonical functions.

In this study, we show that DGCR8 interacted with heterochromatin and nuclear lamina proteins to maintain heterochromatin organization. Expressing a mutant version of DGCR8 lacking the N-terminal region in hMSCs resulted in heterochromatin destabilization and accelerated cellular senescence in a miRNA processing-independent way. Lentiviral-mediated expression of DGCR8, or DGCR8-interacting proteins such as KRAB-associated protein 1 (KAP1), Lamin B1, or HP1γ, rescued senescent phenotypes in DGCR8-deficient hMSCs. Moreover, a decrease in DGCR8 level was detected in hMSCs from older individuals, and restoring level of wildtype DGCR8 (or a miRNA-irrelevant version of DGCR8) attenuated hMSC senescence, as well as post-traumatic articular aging and osteoarthritis in mice. These results demonstrate a non-canonical role for DGCR8 in maintaining nuclear and heterochromatin organization, and protecting against cellular senescence in human MSCs. Thus, DGCR8 represents a new therapeutic target for combating age-associated disorders.

## Results

### Generation of DGCR8-defective human ESCs

**Fig. 1:**
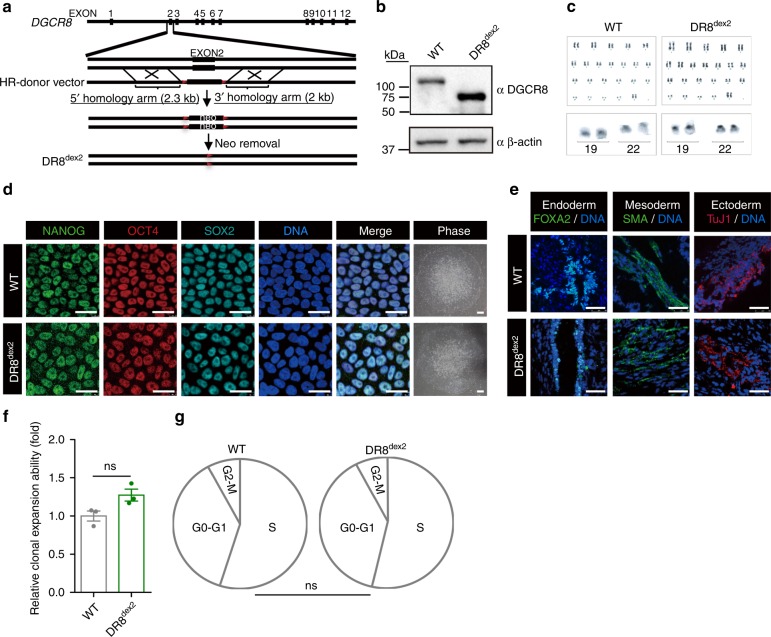
CRISPR/Cas9-mediated gene targeting of DGCR8 in hESCs. **a** Schematic diagram of DGCR8 targeting using CRISPR/Cas9-mediated genome editing with homologous recombination. Neo stands for neomycin-resistance gene cassette used for positive selection. Red triangle indicates the FRT site. **b** Western blotting analysis of DGCR8 with protein extracts from WT and DR8^dex2^ hESCs. β-actin was used as the loading control. **c** G-banded karyotyping analysis of WT and DR8^dex2^ hESCs. **d** Immunofluorescence analysis of pluripotency markers OCT4, SOX2, and NANOG in WT and DR8^dex2^ hESCs. Scale bar, 12.5 μm. **e** Immunofluorescence analysis of representative markers of three germ layers in teratomas derived from WT or DR8^dex2^ hESCs. Scale bar, 50 μm. **f** Clonal expansion analysis of WT and DR8^dex2^ hESCs. Data were presented as mean ± SEM. *n* = 3 wells per cell type, ns means no significance. **g** Cell cycle analysis of WT and DR8^dex2^ hESCs. *n* = 3 wells per cell type, ns means no significance. Statistical significances were assessed by a two-tailed unpaired Student’s *t* test

To study the role of DGCR8, we purposed to generate DGCR8-defective human embryonic stem cells (hESCs) by CRISPR/Cas9-mediated gene editing^22^. Initially, we tried to generate DGCR8-deficient hESCs by targeting exon 3 of *DGCR8* using Cas9 and sgRNAs according to the previous study performed in mice^19^, resulting in DR8^ex3^ hESCs (Supplementary Fig. 1a, b). However, DR8^ex3^ hESCs exhibited abnormal karyotypes, with an inter-chromosomal translocation between chromosomes 19 and 22 (Supplementary Fig. 1c). Therefore, we next sought to target exon 2 of *DGCR8* which contains the start codon ATG (Supplementary Fig. 1d). Complete ablation of full-length DGCR8 protein was confirmed by immunoblotting and immunofluorescence analyses (Supplementary Fig. 1e, p). DR8^ex2^ hESCs were karyotypically normal (Supplementary Fig. 1g), but exhibited poor maintenance of self-renewal capacity (Supplementary Fig. 1f, h–j) and high levels of spontaneous differentiation (Supplementary Fig. 1k). We next generated hESCs with an N-terminal-truncated version of DGCR8 (Deletion of a.a. 1-240) by specifically removing the coding sequence in exon 2 using a CRISPR/Cas9-mediated homologous recombination strategy^23^ (referred to as DR8^dex2^ hESCs) (Fig. 1a, b and Supplementary Fig. 1l, m, p). The resultant DR8^dex2^ hESCs were cultured for more than 50 passages without the emergence of abnormal karyotypes (Fig. 1c), or the loss of pluripotency markers (Fig. 1d and Supplementary Fig. 1n). DR8^dex2^ hESCs were also capable of forming teratomas composed of the three germ layers in vivo, confirming normal pluripotency (Fig. 1e and Supplementary Fig. 1o). Cell cycle kinetics and clonal expansion ability were also normal in DR8^dex2^ hESCs (Fig. 1f, g).

### DGCR8^dex2^ accelerates senescence of hMSCs

**Fig. 2:**
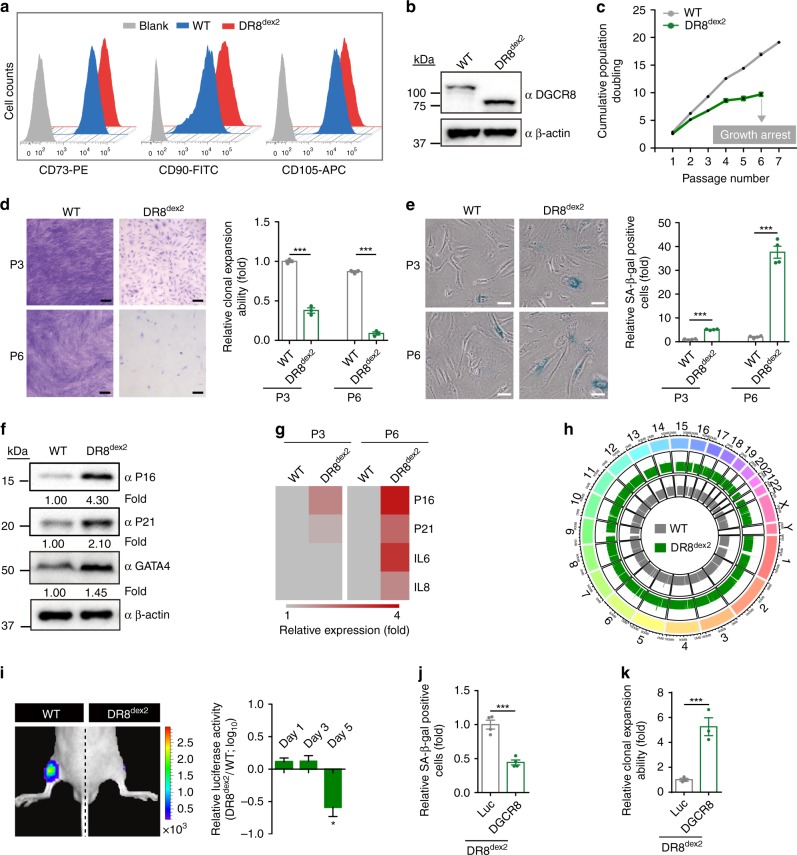
DGCR8^dex2^ accelerates cellular senescence in hMSCs. **a** FACS analysis of the MSC markers CD73, CD90, and CD105 in WT and DR8^dex2^ hMSCs. **b** Western blotting analysis of DGCR8 in WT and DR8^dex2^ hMSCs. β-actin was used as the loading control. **c** Growth curve of WT and DR8^dex2^ hMSCs. **d** Clonal expansion analysis of WT and DR8^dex2^ hMSCs at early passage (P3) and late passage (P6). Scale bar, 30 μm. Data were presented as mean ± SEM. *n* = 3 wells per cell type, ***p < 0.001. **e** SA-β-gal staining of WT and DR8^dex2^ hMSCs at early (P3) and late (P6) passages. Scale bar, 20 μm. Data were presented as mean ± SEM. *n* = 4 images per cell type, ***p < 0.001. **f** Western blotting analysis of P16, P21, and GATA4 in WT and DR8^dex2^ hMSCs. β-actin was used as the loading control. **g** Heatmap showing quantitative RT-PCR analysis of the indicated genes in P3- and P6-hMSCs. Expression levels of the indicated genes in DR8^dex2^ hMSCs were normalized to those in WT hMSCs. (**h**) Whole genome analysis of copy number variations (CNVs) in WT and DR8^dex2^ hMSCs. **i** Photon flux from tibialis anterior (TA) muscles of nude mice transplanted with WT (left) or DR8^dex2^ hMSCs (right) that express luciferase. The attrition of hMSCs after implantation was measured by the reduction of luciferase activity in TA muscles using an in vivo imaging system (IVIS). Data were presented as mean ± SEM. *n* = 3 mice per group, *p < 0.05. **j** SA-β-gal staining of DR8^dex2^ hMSCs transduced with lentiviruses expressing luciferase (Luc) or DGCR8. Data were presented as mean ± SEM. *n* = 4 images per condition, ***p < 0.001. **k** Clonal expansion analysis of DR8^dex2^ hMSCs transduced with lentiviruses expressing Luc or DGCR8. Data were presented as mean ± SEM. *n* = 3 wells per condition, ***p < 0.001. Statistical significances were assessed by a two-tailed unpaired Student’s t test

To characterize the effects of DR8^dex2^ on the function of post-pluripotent cells, we differentiated DR8^dex2^ hESCs into human mesenchymal stem cells (referred to as DR8^dex2^ hMSCs)^9,10,24^. DR8^dex2^ hMSCs were positive for typical hMSC markers, such as CD73, CD90, and CD105, and negative for hMSC-irrelevant antigens, including CD34, CD43, and CD45 (Fig. 2a and Supplementary Fig. 2a). Western blotting verified the lack of full length DGCR8 in DR8^dex2^ hMSCs using an anti-DGCR8 antibody (Fig. 2b). Next, we analyzed the growth rate of DR8^dex2^ hMSCs through serial passaging. Compared with wildtype (WT) hMSCs that showed normal aging kinetics, DR8^dex2^ hMSCs senesced at as early as passage 3, and completely arrested growth at passage 6 (Fig. 2c). In addition to premature senescence, DR8^dex2^ hMSCs exhibited decreased proliferative potential (Fig. 2d and Supplementary Fig. 2b), increased senescence-associated (SA)-β-gal activity (Fig. 2e), upregulation of senescence markers P16 and P21 proteins (Fig. 2f), activation of senescence-associated secretory phenotype (SASP) (Fig. 2g), and enhancement of the DNA damage response (DDR) (Supplementary Fig. 2c, d**)**. Despite increased DDR, genome-wide copy number variation (CNV) analysis indicated that genomic integrity was maintained in DR8^dex2^ hMSCs (Fig. 2h). In line with premature cellular senescence, chondrogenesis and osteogenesis were impaired in DR8^dex2^ hMSCs (Supplementary Fig. 2e, f). Finally, implantation of hMSCs into the tibialis anterior muscles of nude mice (Fig. 2i) revealed accelerated decay of DR8^dex2^ hMSCs compared to WT hMSCs. Taken together, these data suggest that the N-terminal truncation of DGCR8 promotes premature senescence and functional attrition of hMSCs.

### DGCR8^dex2^ exhibits no global effects on miRNA processing

To determine whether the phenotypes of DR8^dex2^ hMSCs resulted from loss or gain of DGCR8 function, WT hMSCs were transduced with lentiviral vectors encoding: 1) Cas9 and a sgRNA targeting exon 5 of *DGCR8*, or 2) a DGCR8-specific shRNA. Both effectively reduced DGCR8 protein levels (Supplementary Fig. 2g, j) and induced premature senescence with phenotypes such as impaired cell proliferation (Supplementary Fig. 2h, k) and early onset of SA-β-gal activity (Supplementary Fig. 2i, l). By contrast, restoring WT DGCR8 (DR8-WT) in DR8^dex2^ hMSCs by lentivirus-mediated cDNA transfer alleviated cellular senescence (Fig. 2j, k and Supplementary Fig. 2m). These observations suggest that accelerated senescence of DR8^dex2^ hMSCs results from loss of DGCR8 activity.

**Fig. 3:**
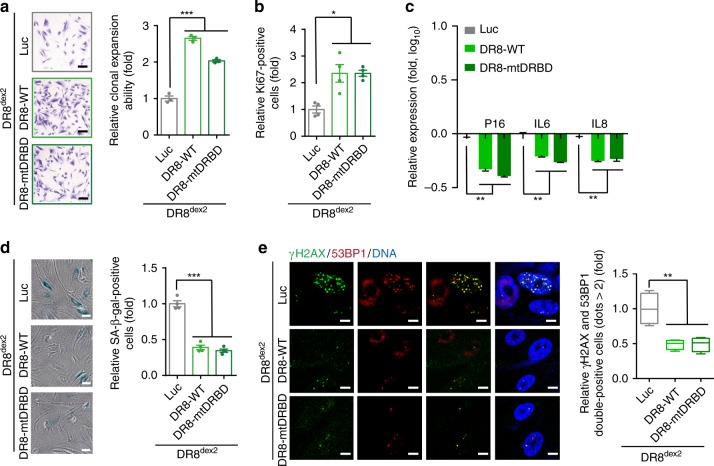
DGCR8 regulates hMSC senescence in a miRNA processing-independent way. **a** Clonal expansion analysis of DR8^dex2^ hMSCs transduced with lentiviruses expressing Luc, DR8-WT, or DR8-mtDRBD. Scale bar, 20 μm. Data were presented as mean ± SEM. *n* = 3 wells per condition, ***p < 0.001. **b** Ki67 staining of DR8^dex2^ hMSCs transduced with lentiviruses expressing Luc, DR8-WT, or DR8-mtDRBD. Data were presented as mean ± SEM. *n* = 4 images per condition, *p < 0.05. **c** RT-qPCR detection of P16, IL6, and IL8 in DR8^dex2^ hMSCs transduced with lentiviruses expressing Luc, DR8-WT, or DR8-mtDRBD. Data were presented as mean ± SEM. *n* = 3 wells per condition, **p < 0.01. **d** SA-β-gal staining of DR8^dex2^ hMSCs transduced with lentiviruses expressing Luc, DR8-WT, or DR8-mtDRBD. Data were presented as mean ± SEM. *n* = 4 images per condition, ***p < 0.001. Scale bar, 20 μm. **e** Immunostaining of γH2AX and 53BP1 in DR8^dex2^ hMSCs transduced with lentiviruses expressing Luc, DR8-WT, or DR8-mtDRBD. Data were presented as mean ± SEM. *n* = 4 images per condition, **p < 0.01. Scale bar, 10 μm. Box plots show the median (center line inside the box), upper and lower quartiles (bounds of box), largest and smallest values (whiskers)

We next investigated whether defective DGCR8 caused accelerated senescence in DR8^dex2^ hMSCs due to loss of its well-known miRNA-processing activity. Consistent with the fact that the C-terminal domains of DGCR8 are necessary for pri-miRNA processing^25^, the interaction between DR8^dex2^ and Drosha was preserved in DR8^dex2^ hMSCs, and there were no global differences in miRNA expression profiles between DR8^dex2^ and WT hMSCs (Supplementary Fig. 3a–c). Further, based on previous studies showing that the simultaneous mutation of amino acids 568, 569, 676, and 677 of DGCR8 abrogate its miRNA-processing ability^25^, we generated an expression vector for DGCR8-AA568-569KK/AS676-677KK (mutant of double-strand RNA-binding domain; mtDRBD), which lacked miRNA-processing function but preserved the interaction with Drosha (Supplementary Fig. 3d, e). Expression of DGCR8 mtDRBD (DR8-mtDRBD) rescued premature aging defects as effectively as DR8-WT, as evidenced by enhanced cell proliferation (Supplementary Fig. 3f and Fig. 3a, b), downregulation of cellular senescence-associated markers (Fig. 3c), reduced SA-β-gal activity (Fig. 3d), decreased DDR (Fig. 3e) in hMSCs-DR8^dex2^. Also, miRNA profiles were not globally changed in DR8^dex2^ hMSCs expressing DR8-WT or DR8-mtDRBD (Supplementary Fig. 3g). This suggests that the miRNA-processing function of DGCR8 is irrelevant to its ability to regulate hMSC senescence.

### DGCR8 is required for maintaining heterochromatin structure

**Fig. 4:**
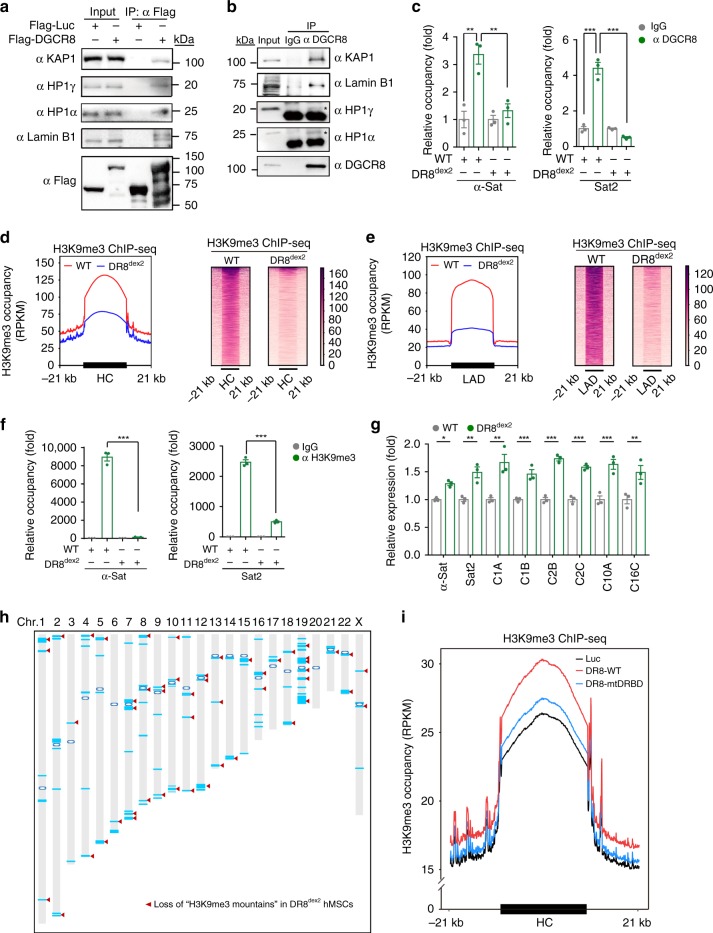
DGCR8 forms a complex with heterochromatin proteins and Lamin B1. **a** Co-immunoprecipitation analysis of KAP1, Lamin B1, HP1α, and HP1γ with exogenous Flag-tagged DGCR8 protein in HEK293T cells. **b** Co-immunoprecipitation analysis of KAP1, Lamin B1, HP1α, and HP1γ with endogenous DGCR8 protein in WT hMSCs. The HP1α and HP1γ bands pulled down by IP with an anti-DGCR8 antibody were indicated by asterisk. **c** Enrichment of DGCR8 within the region of α-Sat or Sat2 measured by ChIP-qPCR. Data were presented as mean ± SEM. *n* = 3 wells per condition, **p < 0.01, ***p < 0.001. **d** Left, enrichment of H3K9me3 ChIP-seq signals ranging from 21 kb upstream to 21 kb downstream of heterochromatin (HC) regions in WT and DR8^dex2^ hMSCs. Right, heatmap showing the enrichment of H3K9me3 signals around HC regions (from 21 kb upstream to 21 kb downstream) in WT and DR8^dex2^ hMSCs. **e** Left, enrichment of H3K9me3 ChIP-seq signals ranging from 21 kb upstream to 21 kb downstream of lamina-associated domain (LAD) regions in WT and DR8^dex2^ hMSCs. Right, heatmap showing the enrichment of H3K9me3 signals around LAD regions (from 21 kb upstream to 21 kb downstream) in WT and DR8^dex2^ hMSCs. **f** Enrichment of H3K9me3 within the region of α-Sat or Sat2 in WT and DR8^dex2^ hMSCs measured by ChIP-qPCR. Data were presented as mean ± SEM. *n* = 3 wells per condition, ***p < 0.001. **g** Quantitative RT-PCR analysis of centromeric element transcripts in WT and DR8^dex2^ hMSCs. Data were presented as mean ± SEM. *n* = 3 wells per condition, *p < 0.05, **p < 0.01, ***p < 0.001. **h** Sketch map of “H3K9me3 mountain” distribution over 23 chromosomes. Blue lines indicate 106 “H3K9me3 mountains” present in WT hMSCs. Red arrowheads indicate 62 “H3K9me3 mountains” lost in DR8^dex2^ hMSCs. Blue circles indicate the centromeres of chromosomes. **i** Enrichment of H3K9me3 ChIP-seq signals ranging from 21 kb upstream to 21 kb downstream of heterochromatin (HC) regions in DR8^dex2^ hMSCs transduced with Luc, DR8-WT, or DR8-mtDRBD. Statistical significances were assessed by a two-tailed unpaired Student’s *t* test

To further explain why N-terminal truncation of DGCR8 induced accelerated MSC senescence, we sought to identify new DGCR8-interacting proteins. We expressed Flag-tagged DGCR8 in HEK293T cells and performed immunoprecipitation with an antibody against Flag, followed by mass spectrometry (Supplementary Fig. 4a). In addition to well-known DGCR8-associated proteins like Drosha, we identified a component of heterochromatin, KAP1 (Supplementary Fig. 4b). Due to the limited resolution of mass spectrometry in recognizing low abundant and trypsin-insensitive proteins^9^, we performed co-immunoprecipitation (Co-IP) and identified more heterochromatin components that interacted with DGCR8, revealing DGCR8 as part of a complex containing major heterochromatin proteins KAP1, HP1α, HP1γ, as well as the nuclear lamina protein Lamin B1 (Fig. 4a, b and Supplementary Fig. 4c). In addition, chromatin immunoprecipitation-quantitative polymerase chain reaction (ChIP-qPCR) revealed that DGCR8 bound pericentromeric repetitive sequences, including α-Satellite (α-Sat) and Satellite 2 (Sat2) (Fig. 4c), which are primarily wrapped as architecturally condensed constitutive heterochromatin^26–28^. Furthermore, DGCR8 associated with heterochromatin-enriched genomic regions such as LINE1 and ribosomal DNA (rDNA)^4,10,29–31^ (Supplementary Fig. 4d). These data indicate that DGCR8 may be an important component of constitutive heterochromatin.

In DR8^dex2^ hMSCs, expression levels of KAP1, HP1α, HP1γ, and Lamin B1 were each decreased (Supplementary Fig. 4e). ChIP sequencing (ChIP-seq) analysis of DR8^dex2^ hMSCs revealed the downregulation of H3K9me3, a marker of constitutive heterochromatin, across the genome, especially within heterochromatin (HC) and lamina-associated domains (LADs) (Fig. 4d, e and Supplementary Fig. 5a–c). ChIP-qPCR further confirmed reduced H3K9me3 enrichment at genomic repetitive sequences, including α-Sat, Sat2, LINE1, and rDNA loci (Fig. 4f and Supplementary Fig. 4f), correlating with the upregulation of transcripts from these repetitive sequences (Fig. 4g and Supplementary Fig. 4g). In addition, we observed loss of “H3K9me3 mountains” in DR8^dex2^ hMSCs, which were mainly located at genomic repetitive sequences, such as pericentromeres (Fig. 4h and Supplementary Fig. 5d). Consistent with these changes in chromatin architecture, RNA-seq analysis revealed global changes in gene expression associated with heterochromatin decondensation in DR8^dex2^ hMSCs (Supplementary Fig. 6a–c). Finally, these heterochromatin alterations were reversed by the re-introduction of DR8-WT or DR8-mtDRBD, which was demonstrated by H3K9me3 ChIP-seq (Fig. 4i and Supplementary Fig. 6d). Taken together, these data indicate that DGCR8 is essential for maintaining heterochromatin architecture.

### DGCR8 interacts with Lamin B1 and HC components

**Fig. 5:**
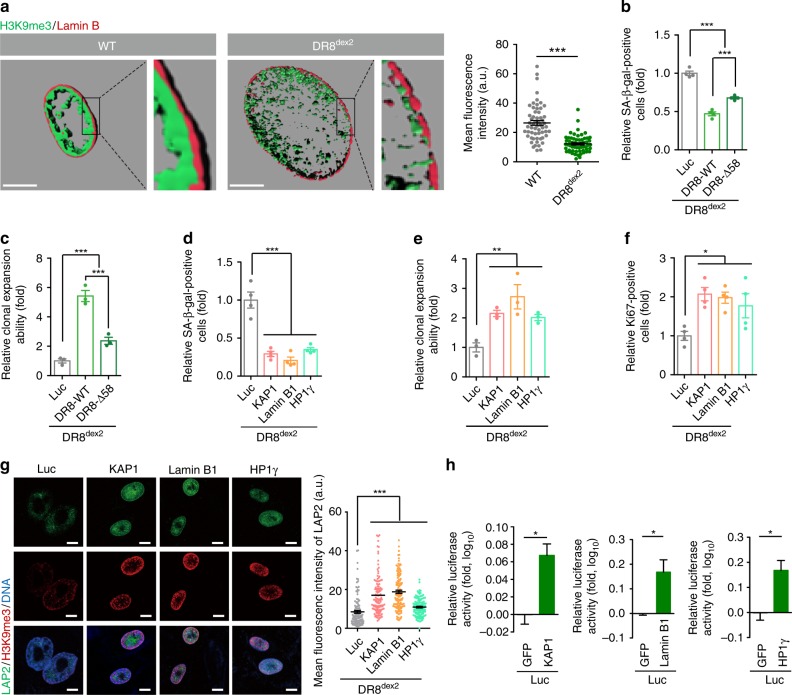
DGCR8 and its interacting proteins are required for the homeostatic maintenance of hMSCs. **a** Left, 3D construction of a z stack of H3K9me3 and Lamin B immunofluorescence images shown in supplementary Fig. 7i of WT and DR8^dex2^ hMSCs. Scale bar, 10 μm. Right, mean fluorescence intensity of H3K9me3 in supplementary Fig. 7i was measured by ImageJ. Data were presented as mean ± SEM. *n* = 60 cells per cell type, ***p < 0. 001. **b** SA-β-gal staining of DR8^dex2^ hMSCs transduced with lentiviruses expressing Luc, DR8-WT (1-773), or DR8-Δ58. Data were presented as mean ± SEM. *n* = 4 images per condition, ***p < 0.001. **c** Clonal expansion analysis of DR8^dex2^ hMSCs transduced with lentiviruses expressing Luc, DR8-WT (1-773), or DR8-Δ58. Data were presented as mean ± SEM. *n* = 3 wells per condition, ***p < 0.001. **d** SA-β-gal staining of DR8^dex2^ hMSCs transduced with lentiviruses expressing Luc, KAP1, Lamin B1, or HP1γ. Data were presented as mean ± SEM. *n* = 4 images per condition, ***p < 0.001. **e** Clonal expansion analysis of DR8^dex2^ hMSCs transduced with lentiviruses expressing Luc, KAP1, Lamin B1, or HP1γ. Data were presented as mean ± SEM. *n* = 3 wells per condition, **p < 0.01. **f** Ki67 staining of DR8^dex2^ hMSCs transduced with lentiviruses expressing Luc, KAP1, Lamin B1, or HP1γ. Data were presented as mean ± SEM. *n* = 4 images per condition, *p < 0.05. **g** Immunostaining of LAP2 and H3K9me3 in DR8^dex2^ hMSCs transduced with lentiviruses expressing Luc, KAP1, Lamin B1, or HP1γ. Scale bar, 10 μm. Mean fluorescence intensity of LAP2 was calculated. Data were presented as mean ± SEM. *n* = 120 cells per condition. ***p < 0.001. **h** Photon flux from tibialis anterior (TA) muscles of nude mice transplanted with DR8^dex2^ hMSCs with co-infection of lentiviral vectors expressing GFP or KAP1 (left)/Lamin B1 (middle)/HP1γ (right) and lentiviral vector expressing luciferase. Luciferase activity in the TA muscles was detected by an in vivo imaging system (IVIS) 4 days after implantation, demonstrating slower attrition of DR8^dex2^ hMSCs transduced with KAP1, Lamin B1, or HP1γ. Data were presented as mean ± SEM. *n* = 3 mice per group, *p < 0.05. Statistical significances were assessed by a two-tailed unpaired Student’s t test

In human premature aging disorders, enlarged nuclei and loss of heterochromatin underneath the nuclear envelope are often observed^9^. Similar features were present in DR8^dex2^ hMSCs (Fig. 5a and Supplementary Fig. 7a, b, and i), suggesting a causative link between the inability of DR8^dex2^ to maintain heterochromatin organization and accelerated cellular senescence.

To map the domains of DGCR8 required for interacting with Lamin B1 and heterochromatin components, we generated a series of Flag-tagged DGCR8 fragments, including a.a. 1–300, a.a. 1–510, a.a. 300–773, a.a. 358–773, and a.a. 510–773 (Supplementary Fig. 7c–e). DGCR8 a.a. 300–773 was excluded from further investigations due to cytoplasmic mislocalization (Supplementary Fig. 7e). Both DGCR8 a.a. 1–300 and a.a. 510–773 contain domains that interacted with the heterochromatin components, KAP1 and HP1γ. For the a.a. 1–510 construct, a.a. 300–510 exerted an auto-inhibitory effect on a.a. 1–300 (Supplementary Fig. 7g). A docking site for Lamin B1 was deduced in DGCR8 a.a. 300–358 (Supplementary Fig. 7g, h). These studies suggest that DGCR8 acts as a scaffold protein to tether heterochromatin proteins to the nuclear lamina. Subsequently, we investigated whether such a scaffold-like activity of DGCR8 is required for regulating cellular senescence. We generated a DGCR8 mutant (DR8-Δ58, which lacks a.a. 300–358) that interacted with KAP1 and HP1γ, but not Lamin B1 (Supplementary Fig. 7f, h). Compared to DR8-WT, DR8-Δ58 was less able to rescue accelerated senescence in DR8^dex2^ hMSCs (Fig. 5b, c). On the other hand, overexpression of Lamin B1, KAP1, or HP1γ each alleviated senescent phenotypes of DR8^dex2^ hMSCs both in vitro and in vivo (Fig. 5d–h and Supplementary Fig. 8a, b). Taken together, these data suggest that DGCR8 regulates hMSC aging by maintaining heterochromatin organization via its interactions with Lamin B1 and heterochromatin components.

### DGCR8 overexpression exerts a geroprotective role

**Fig. 6:**
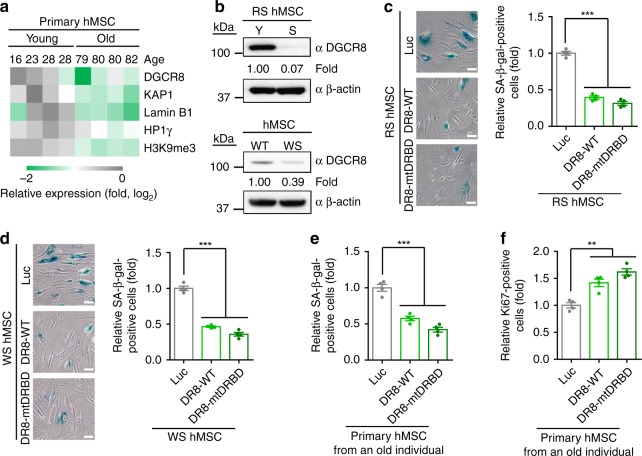
DGCR8 is downregulated in senescent hMSCs and DGCR8 overexpression attenuates hMSC aging. **a** Heatmap showing the quantification of protein levels in young and old human primary MSCs. Expression levels of indicated proteins in old hMSCs were normalized to those in young hMSCs. **b** Western blotting analysis of DGCR8 in replicative senescent (RS) and WS hMSCs. Y, young, S, senescent. β-actin was used as the loading control. **c** SA-β-gal staining of RS hMSCs transduced with Luc, DR8-WT, or DR8-mtDRBD. Data were presented as mean ± SEM. *n* = 4 images per condition, ***p < 0.001. Scale bar, 20 μm. **d** SA-β-gal staining of WS hMSCs transduced with Luc, DR8-WT, or DR8-mtDRBD. Data were presented as mean ± SEM. *n* = 4 images per condition, ***p < 0.001. Scale bar, 20 μm. **e** SA-β-gal staining of primary hMSCs transduced with lentiviruses expressing Luc, DR8-WT, or DR8-mtDRBD. Data were presented as mean ± SEM. *n* = 4 images per condition, ***p < 0.001. **f** Ki67 staining of primary hMSCs transduced with lentiviruses expressing Luc, DR8-WT, or DR8-mtDRBD. Data were presented as mean ± SEM. *n* = 4 images per condition, **p < 0.01. Statistical significances were assessed by a two-tailed unpaired Student’s *t* test

We next investigated DGCR8 levels during physiological and pathological aging. In primary hMSCs derived from human donors at different ages, DGCR8 levels decreased with age (Fig. 6a and Supplementary Fig. 9a). DGCR8 was also downregulated in three stem cell models of human aging, including replicative senescent hMSCs (RS hMSCs), WRN-deficient hMSCs (WS-specific hMSCs) and *LMNA* (p.G608G)-mutated hMSCs (HGPS-specific hMSCs) (Fig. 6b and Supplementary Fig. 9b).

We then explored the possibility of targeting DGCR8 to alleviate human cellular aging. We transduced hMSCs with lentiviruses expressing DR8-WT or DR8-mtDRBD which could not participate in miRNA processing, and assessed aging phenotypes. Overexpression of either form of DGCR8 effectively rescued premature aging phenotypes in RS hMSCs (Fig. 6c and Supplementary Fig. 9c–e, j) and WS-specific hMSCs (Fig. 6d and Supplementary Fig. 9f, g, j). The senescence-associated phenotypes of hMSCs isolated from an 80-year-old individual were also alleviated by overexpressing either DR8-WT or DR8-mtDRBD (Fig. 6e, f and Supplementary Fig. 9h–j**)**.

**Fig. 7:**
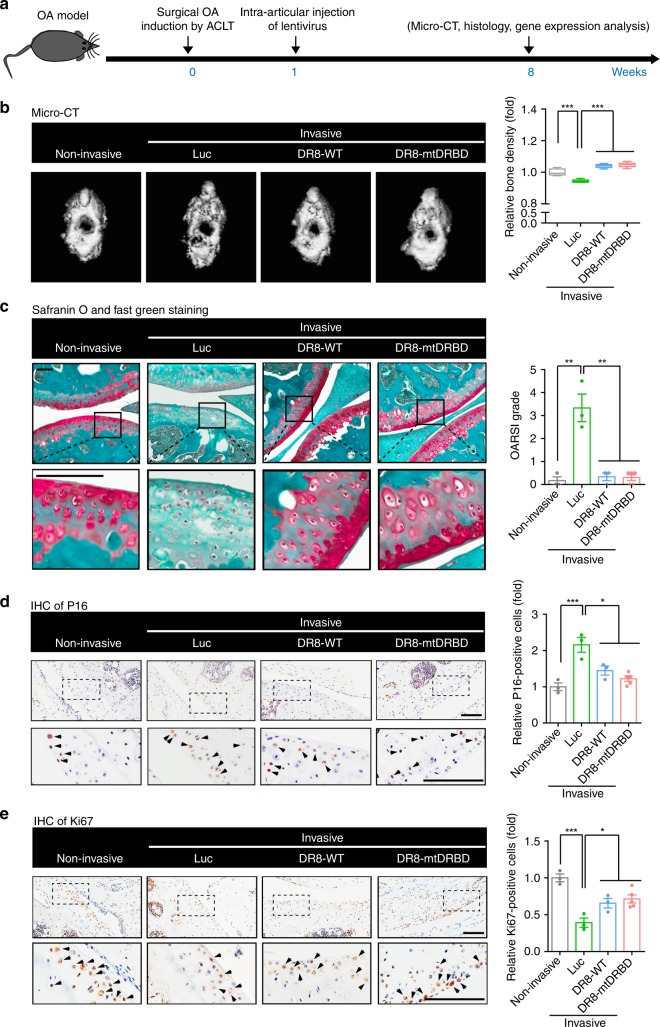
DGCR8 alleviates mouse osteoarthritis. **a** Schematic of the time course for experiments in **b**–**e**. **b** Bone density analysis of mouse joints (Non-invasive, *n* = 3 mice; Luc, *n* = 3 mice; DR8-WT, *n* = 3 mice; DR8-mtDRBD, *n* = 5 mice). Data were presented as mean ± SEM. ***p < 0.001. Box plots show the median (center line inside the box), upper and lower quartiles (bounds of box), largest and smallest values (whiskers). **c** Left, Representative images showing Safranin O and Fast Green staining of articular cartilage from the joints of non-invasive mice (*n* = 3 mice) and ACLT mice injected with lentiviruses expressing Luc (*n* = 3 mice), DR8-WT (*n* = 3 mice), or DR8-mtDRBD (*n* = 5 mice). Right, OARSI score analysis of articular cartilage. Scale bar, 100 μm. Data were presented as mean ± SEM. **p < 0.01. **d** Left, Representative images showing P16 immunohistochemistry of articular cartilage from the joints of non-invasive mice (*n* = 3 mice) and ACLT mice injected with lentiviruses expressing Luc (*n* = 3 mice), DR8-WT (*n* = 3 mice), or DR8-mtDRBD (*n* = 5 mice). Right, Statistical analysis of P16-positive cells. Scale bar, 100 μm. Data were presented as mean ± SEM. *p < 0.05, ***p < 0.001. **e** Left, Representative images showing Ki67 immunohistochemistry of articular cartilage from the joints of non-invasive (*n* = 3 mice) and ACLT mice injected with lentiviruses expressing Luc (*n* = 3 mice), DR8-WT (*n* = 3 mice), or DR8-mtDRBD (*n* = 5 mice). Right, Statistical analysis of Ki67-positive cells. Scale bar, 100 μm. Data were presented as mean ± SEM. *p < 0.05, ***p < 0.001. Statistical significances were assessed by a two-tailed unpaired Student’s t test

**Fig. 8:**
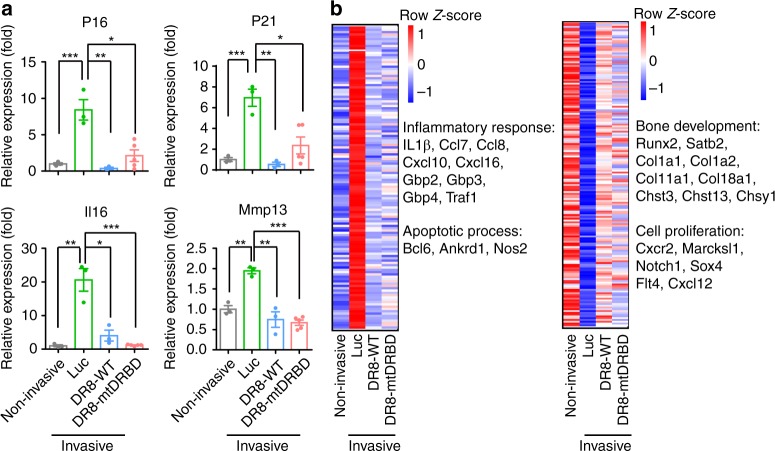
Aging and inflammation factors dysregulated in mouse osteoarthritis are rescued by DGCR8 overexpression. **a** Quantitative RT-PCR analysis of P16, P21, Il6 and Mmp13 in non-invasive mouse (*n* = 3 mice) joints and ACLT joints treated with lentiviruses expressing Luc (*n* = 3 mice), DR8-WT (*n* = 3 mice) or DR8-mtDRBD (*n* = 5 mice). Data were presented as mean ± SEM. *p < 0.05, **p < 0.01, ***p < 0.001. **b** Left, heatmap showing Z-score normalized expression levels of downregulated genes in non-invasive mouse joints and ACLT joints treated with lentiviruses expressing DR8-WT or DR8-mtDRBD, compared with ACLT joints treated with lentiviruses expressing Luc. Right, heatmap showing Z-score normalized expression levels of upregulated genes in non-invasive mouse joints and ACLT joints treated with lentiviruses expressing DR8-WT or DR8-mtDRBD, compared with ACLT joints treated with lentiviruses expressing Luc. The downregulated and upregulated genes were listed in Supplementary Data 7. Statistical significances were assessed by a two-tailed unpaired Student’s *t* test

**Fig. 9:**
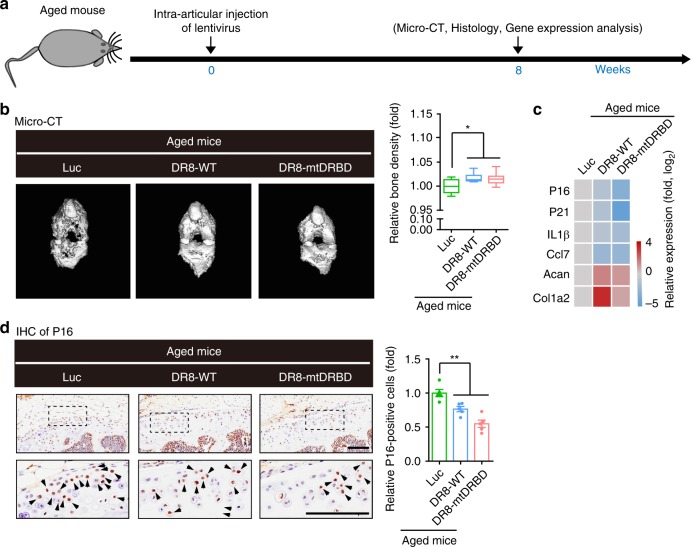
DGCR8 alleviates osteoarthritis of aged mice. **a** Schematic of the time course for experiments in **b**–**d**. **b** Bone density analysis of mouse joints. Data were presented as mean ± SEM. *n* = 5 mice per group, *p < 0.05. Box plots show the median (center line inside the box), upper and lower quartiles (bounds of box), largest and smallest values (whiskers). **c** Heatmap showing quantitative RT-PCR analysis of the indicated genes in aged mouse joints with lentivirus treatment. Expression levels of the indicated genes in aged mouse joints were normalized to Luc group. **d** Left, Immunohistochemistry of P16 expression in aged mouse joints with lentivirus treatment. Right, Statistical analysis of P16-positive cells. Scale bar, 100 μm. Data were presented as mean ± SEM. *n* = 5 mice per group, **p < 0.01. Statistical significances were assessed by a two-tailed unpaired Student’s *t* test

Lastly, we evaluated the in vivo beneficial effects of overexpressing DGCR8 on alleviating aging-related phenotypes. Joint is a critical tissue affected by the accumulation of senescent cells, which serves as a key inducement for the development of osteoarthritis^32^. Recently, the chemical clearance of senescent cells in mouse bone joint has been shown to alleviate the phenotypes of post-traumatic osteoarthritis and even to prevent age-associated bone loss^33^. Accordingly, we proposed that the ectopic expression of DGCR8 may attenuate the development of osteoarthritis by repressing cellular senescence. We utilized anterior cruciate ligament transection (ACLT) to induce articular aging and osteoarthritis in mice^33^ (Fig. 7a**)**. One week after ACLT, lentiviruses encoding luciferase (Luc), DR8-WT or DR8-mtDRBD were injected into the articular cavities of these mice. Seven weeks post lentivirus injection, microcomputed tomography (micro-CT) analyses showed that in contrast to the Luc-injected ACLT mice that exhibited obvious bone damage, the bone quality of the ACLT mice with DR8-WT or DR8-mtDRBD was well maintained as those of the mice without ACLT (Fig. 7b**)**. The subsequent histological assessment showed that ACLT-induced articular cartilage degeneration as the classical phenotype of osteoarthritis was rescued by injecting lentiviruses encoding DR8-WT or DR8-mtDRBD (Fig. 7c). Cellular senescence marker P16 was decreased whereas cellular proliferation marker Ki67 was increased in the articular cartilage of ACLT mice treated with lentiviral vectors expressing DR8-WT or DR8-mtDRBD as indicated by immunohistochemistry (Fig. 7d, e). In addition, the mRNA levels of typical aging markers (e.g., P16 and P21) and inflammation factors (e.g., IL6 and matrix metalloproteinase 13 (MMP13)) that are often upregulated in osteoarthritis were diminished in the articular cavities of the mice with DR8-WT or DR8-mtDRBD (Fig. 8a**)**. Furthermore, RNA-seq analysis revealed that the ectopic expression of DR8-WT or DR8-mtDRBD reversed major gene expression alterations including increased inflammation and apoptosis, and also promoted osteogenesis and chondrogenesis in the articular cavities of the mice with ACLT-induced osteoarthritis (Fig. 8b and Supplementary Fig. 10a, b). Meanwhile, the rescue effect of DGCR8 on osteoarthritis was further evaluated in physiologically aged mice (Fig. 9a**)**. Similar to ACLT mice, physiologically aged mice exhibited decreased bone density, upregulated aging markers and inflammation factors, and downregulated bone development-associated genes (Supplementary Fig. 10c–e). The bone quality of aged mice treated with lentiviral vectors expressing DR8-WT or DR8-mtDRBD was obviously improved compared to luc-expressed lentivirus-treated mice as control (Fig. 9b**)**. In addition, the expression levels of factors involved in osteoarthritis were also rescued by DR8-WT or DR8-mtDRBD lentivirus-mediated treatment (Fig. 9c, d). Taken together, these results demonstrate that the inhibitory effects on cellular senescence and inflammatory response by DGCR8 may contribute to regeneration of bone and cartilage and alleviation of the symptoms of age-related osteoarthritis, suggesting the therapeutic potential of DGCR8 as a geroprotective factor.

## Discussion

In this study, we presented several lines of evidence supporting a non-canonical, miRNA processing-independent role of DGCR8 in regulating heterochromatin organization and protecting against cellular aging. First, DGCR8 was essential for preventing premature senescence by maintaining heterochromatin organization via interactions with both heterochromatin and lamina proteins. Second, DGCR8 levels were downregulated with age. Third, DGCR8 was geroprotective, both in vitro and in vivo. For the first time, we show that DGCR8 regulated hMSC aging through heterochromatin maintenance, thus identifying DGCR8 as an innovative intervention target for aging.

DGCR8 has been well studied as a miRNA-processing protein^19,34–36^. DGCR8-knockout mESCs arrest in the G1 phase of the cell cycle, a phenotype that can be rescued by members of the miR-290 family^35^. Disrupting DGCR8 in vascular smooth muscle cells reduces cell proliferation and promotes apoptosis via the downregulation of miR-17/92 and miR-143/145 clusters^34^. Conditional knockout of DGCR8 in skin results in the depletion of miRNAs and skin developmental defects^36^. In addition, DGCR8 regulates neural development in both miRNA-dependent and miRNA-independent ways^37,38^. A recent study has also reported a miRNA-independent function of DGCR8 in facilitating the splicing of Tcf711 that is necessary for mESCs to exit pluripotency and differentiate^39^. This suggests that DGCR8 has miRNA-independent functions. Here, we demonstrate that DGCR8 plays a distinct role in regulating hMSC senescence due to its function in maintaining heterochromatin architecture rather than miRNA processing.

We observed that hMSCs with loss of DGCR8 function exhibited abnormal nuclear and chromatin organization, with no marked changes in global miRNA profiles. Although 10.9% of the total miRNAs were differentially expressed in DR8^dex2^ hMSCs, these miRNAs may not be the major contributor to the heterochromatin changes in DR8^dex2^ hMSCs as evidenced by the minimal changes in miRNA profiles of DR8^dex2^ hMSCs expressing Luc, DR8-WT, or DR8-mtDRBD. To our knowledge, this is the first evidence that DGCR8 stabilizes heterochromatin by interacting with the nuclear lamina and heterochromatin proteins, independent of its miRNA-processing ability. We further propose a model in which DGCR8 functions as a scaffold protein to organize heterochromatin by linking heterochromatin with the nuclear lamina (Supplementary Fig. 11). Interestingly, deficiencies in Dicer, which processes pre-miRNAs into mature miRNAs, also induce heterochromatin disorganization and increase genomic instability in *Drosophila*^40^. In contrast to the protein-protein interaction between DGCR8 and heterochromatin components that we report in this study, deficiencies in Dicer disrupt localization of H3K9me2 by altering the specificity of siRNA-mediated targeting of H3K9me2. Despite utilizing different mechanisms, these two studies demonstrate the long-overlooked, non-canonical roles of these two miRNA-processing proteins in regulating nuclear and chromatin organization.

Previous studies on DGCR8 have primarily focused on its miRNA-processing function using DGCR8-knockout cell lines and animal models. DGCR8-deficient mouse fibroblasts and neural stem cells (NSCs) can be reprogrammed into iPSCs with lower efficiency due to the lack of canonical miRNAs^41^. Consistently, iPSCs derived from DGCR8-deficient fibroblasts and NSCs exhibit poor differentiation potentials, similar to DGCR8-deficient mESCs, which fail to downregulate pluripotency markers and therefore do not differentiate^19^. While all of these studies indicate that DGCR8 is required for the self-renewal and differentiation of mouse pluripotent stem cells in a miRNA-dependent manner, the potential for DGCR8 to regulate physiological functions in human stem cells remains unclear. In our multiple efforts to generate human ESC lines with DGCR8 defects for studying the novel functions of DGCR8, DR8^ex2^ hESCs with complete protein ablation failed to maintain pluripotency, and DR8^ex3^ hESCs, which expressed a 50-kDa truncated protein, exhibited genomic instability (primarily an intra-chromosomal translocation). However, DR8^dex2^ hESCs, which lacked the N-terminus of DGCR8, had normal self-renewal ability and pluripotency, but compromised proliferation upon differentiation into hMSCs. The diverse characteristics related to different DGCR8 defects suggest that the regulatory activities by DGCR8 are cell-type and developmental-stage dependent.

With previous studies having mainly utilized mouse cell lines, our study employed the CRISPR/Cas9-mediated gene editing techniques in human ESCs, followed by the directed differentiation of these ESCs to elucidate how DGCR8 regulates the homeostasis and senescence of adult stem/progenitor cells. Genetically modified DR8^dex2^ hESCs have the potential to self-renew, to differentiate into various adult stem cells and further into terminally differentiated cells. Previous studies have shown that the C-terminal domains of DGCR8 mainly participate in miRNA processing. We used DR8^dex2^ hMSCs to reveal that an intact DGCR8 N-terminus is required for heterochromatin organization and to protect against premature senescence. Strikingly, functional inactivation of DGCR8 in DR8^dex2^ hMSCs could be phenotypically rescued by expressing DR8-mtDRBD, which was deficient in miRNA processing. Moving forward, DR8^dex2^ hESCs provide an invaluable platform for investigating the cell type-specific consequences of DGCR8 in human aging-associated disorders, with implications in induced neural cells, cardiomyocytes, and vascular cells, etc., without the confounding effects of DGCR8-associated miRNA clusters.

We and others have recently provided evidence that disorganization of the nuclear envelope and heterochromatin is a driving force for hMSC aging, and that these aging processes can be targeted either by gene correction of disease-associated mutations, by gene editing-mediated cellular enhancement, or by treatment with geroprotective compounds^4,7,9,15,24,42^. In this study, we demonstrate that DGCR8 is a key regulator of human cellular aging via a heterochromatin-stabilizing mechanism. DGCR8 was downregulated in prematurely and physiologically aging hMSCs, and DGCR8 overexpression effectively alleviated senescent phenotypes. Notably, the accumulation of senescent cells and their secretion of SASP factors in many tissues are considered as risk factors for age-related pathologies, like osteoarthritis^33,43^. Due to the complexity and heterogeneity of osteoarthritis pathogenesis, the combined removal of local senescent cells and the transplantation of MSCs may be a good therapeutic option to date^33,43–45^. Here, we provide strong in vivo evidence that osteoarthritic mice that received gene therapy (using lentiviruses encoding DR8-WT or DR8-mtDRBD) exhibited reduced inflammation and increased osteogenesis- and chondrogenesis-related gene expression, suggesting enhanced regeneration in the articular cavity, and effective restoration of cartilage structure. Thus, this is a promising strategy for treating age- or injury-associated osteoarthritis. Given the fact that DR8-mtDRBD does not affect miRNA processing, this modified protein may represent a robust and safer gene therapeutic tool for treating osteoarthritis. Taken together, our study provides the first evidence that DGCR8 is a gatekeeper for maintaining heterochromatin organization, highlighting the fact that DGCR8-heterochromatin may be a novel target for understanding and hopefully treating aging-associated disorders in the future.

## Methods

### Cell culture

Wildtype (WT) human ESCs (hESCs, Line H9, WiCell Research Institute) and their genetic modified derivatives were maintained on feeder cells (mitomycin C-inactivated mouse embryonic fibroblast, MEF) in hESC culture medium containing DMEM/F12 (Thermo Fisher Scientific), 20% Knockout Serum Replacement (Thermo Fisher Scientific), 0.1 mM non-essential amino acids (NEAA, Thermo Fisher Scientific), 2 mM GlutaMAX (Thermo Fisher Scientific), 55 μM β-mercaptoethanol (Thermo Fisher Scientific), and 10 ng/ml bFGF (Joint Protein Central) or on Matrigel (BD Biosciences) in mTeSR medium (STEMCELL Technologies). Both Human MSCs (hMSCs) derived from hESCs and primary human MSCs were cultured in MEMα (Thermo Fisher Scientific) medium supplemented with 10% fetal bovine serum (FBS, Thermo Fisher Scientific (Cat:10099-141, Lot:1616964)), 0.1 mM non-essential amino acids (Thermo Fisher Scientific), 1% penicillin/streptomycin (Thermo Fisher Scientific), and 1 ng/ml bFGF (Joint Protein Central, JPC).

### Antibodies

Antibodies used for western blotting included anti-DGCR8 (Ab191875, Abcam, 1:1000), anti-KAP1 (Ab22553, Abcam, 1:2000), anti-Lamin B1 (Ab16048, Abcam, 1:1000), anti-HP1α (#2616 S, Cell signaling, 1:1000), anti-HP1γ (#2619, Cell signaling, 1:3000), anti-LAP2 (611000, BD Biosciences, 1:1000), anti-P16 (550834, BD Biosciences, 1:500), anti-P21 (2947, Cell signaling, 1:1000), anti-GATA4 (sc-9053, SCBT, 1:1000), anti-HDAC1 (sc-7872, SCBT, 1:1000), anti-LBR (Ab32535, Abcam, 1:1000), anti-SUV39H1 (05615, Millipore, 1:1000), anti-Drosha (Ab12286, Abcam, 1:1000), anti-β-actin (sc-69879, SCBT, 1:5000), anti-GAPDH (sc-25778, SCBT, 1:3000). Antibodies used for immunostaining included anti-DGCR8 (Ab191875, Abcam, 1:1000), anti-Ki67 (VP-RM04, Vector, 1:800), anti-γH2AX (05636, Millipore, 1:400), anti-53BP1 (A300-273A, Bethyl Laboratories, 1:500), anti-FOXA2 (8186 S, Cell signaling, 1:100), anti-SMA (A5228, Sigma Aldrich, 1:100), anti-TuJ1 (T2220, Sigma Aldrich, 1:100), anti-H3K9me3 (Ab8898, Abcam, 1:800), anti-Lamin B (sc-6217, SCBT, 1:250), anti-LAP2 (611000, BD Biosciences, 1:500), anti-OCT3/4 (sc-5279, SCBT, 1:200), anti-SOX2 (sc-17320, SCBT, 1:100), anti-NANOG (Ab21624, Abcam, 1:200). Antibodies used for flow cytometry included anti-CD105 (17-1057-42, eBioscience, 1:100), anti-CD73 (550257, BD Biosciences, 1:100), anti-CD90 (555595, BD Biosciences, 1:100), anti-CD34 (555822, BD Biosciences, 1:100), anti-CD43 (580198, BD Biosciences, 1:100), anti-CD45 (555482, BD Biosciences, 1:100). Antibodies used for IHC included anti-P16 (Ab54210, Abcam, 1:1000), anti-Ki67 (GB13030-2, Servicebio, 1:500).

### CRISPR/Cas9-mediated *DGCR8* editing in hESCs

CRISPR/Cas9-mediated gene editing was performed as previously described with modifications^22^. In brief, guide RNAs (gRNAs) were designed at the website http://crispr.mit.edu. The coding sequences of gRNAs targeting exon 2 or exon 3 of *DGCR8* were cloned into pCAG-mCherry-gRNA^46^. Upon treatment with ROCK inhibitor (Y-27632, TOCRIS) for 24 h, hESCs were electroporated by 4D-Nucleofector (Lonza) with both a gRNA vector and pCAG-1BPNLS-Cas9-1BPNLS-2AGFP^46^. Cells were then seeded on Matrigel-coated plates and cultured with mTeSR medium. After expansion for 48 h, EGFP/mCherry-dual-positive cells were collected by FACS and cultured on MEF feeders in hESC culture medium. Clones were manually picked and expanded for further characterization.

### CRISPR/Cas9-mediated generation of DR8^dex2^ hESCs

CRISPR/Cas9-mediated gene editing was performed as previously described with modifications^47^. In brief, a donor plasmid was constructed with two 1.0–2.0-kb homology arms and a drug resistance cassette (neomycin). A gRNA targeting exon 2 of *DGCR8* was cloned into a gRNA cloning vector (#41824, Addgene) (DGCR8-gRNA). Upon treatment with ROCK inhibitor (Y-27632, TOCRIS) for 24 hours, hESCs (5 × 10^6^) were resuspended in 100 μl opti-MEM (Thermo Fisher Scientific) containing 7 μg hCas9 (#41815, Addgene), 7 μg DGCR8-gRNA and 7 μg donor plasmid and electroporated by 4D-Nucleofector (Lonza). Cells were then seeded on MEF feeder cells. G418 (100 μg/ml, Thermo Fisher Scientific) was added to initiate positive selection 2–4 days after electroporation. After G418 selection and clonal expansion for about 2 weeks, G418-resistant clones were picked and transferred to a 96-well plate for further characterization and expansion. The identification of gene-targeted clones was performed by genomic PCR and western blotting. Additionally, the pCAG-FLpo-2A-puro vector was used to remove the neomycin-resistance cassette in DR8 ^dex2^ hESCs. Three days after the transfection, puromycin (1 μg/ml, Thermo Fisher Scientific) was used to enrich puro-resistant cells for 48 h. Ten days later, the emerging colonies were picked and expanded for future application.

### Lentiviral CRISPR/Cas9-mediated *DGCR8* or *LMNB1* knockdown

Lentiviral CRISPR/Cas9-mediated gene editing was conducted as previously described^48,49^. Briefly, the sgRNA targeting *DGCR8* or *LMNB1 *was cloned into lenti-CRISPRv2 (#52961, Addgene) with an hSpCas9 expression cassette (pLenti-CRISPRv2-DGCR8 or pLenti-CRISPRv2-LMNB1). Lentiviruses were then packaged with pLenti-CRISPRv2-DGCR8 or pLenti-CRISPRv2-LMNB1 and used to transduce WT hMSCs. Cells were treated with 1 μg/ml puromycin (Thermo Fisher Scientific) for screening at 72 h after the infection.

### Generation and characterization of hMSCs

hMSCs were differentiated from hESCs as previously described^24,50^. Briefly, embryoid bodies were plated on Matrigel-coated plates in hMSC differentiation medium (MEMα (Thermo Fisher Scientific), 10% fetal bovine serum (FBS, Thermo Fisher Scientific (Cat:10099-141, Lot:1616964)), 1% penicillin/streptomycin (Thermo Fisher Scientific), 1 ng/mL bFGF (Joint Protein Central) and 5 ng/mL TGFβ (Humanzyme)) for 10 days. The confluent fibroblast-like cells were then maintained in hMSC culture medium and FACS-sorted (BD FACS Aria II) for the purification of CD73/CD90/CD105 tri-positive hMSCs. The functionality of hMSCs was verified by differentiation to osteoblasts, chondrocytes, and adipocytes of the sorted hMSCs^24,50^.

### Isolation and culture of primary hMSCs

Primary hMSCs were isolated from the dental pulp of different individuals with the approval from the Ethics Committee of the 306 Hospital of PLA in Beijing^44^. The tissue block was cut using scissors in 1x TrypLE™ Express Enzyme plus Dispase IV and further digested at 37 °C for 30 min. The digestion mixture was passed through a 100 μm cell strainer and centrifuged at 500 g for 10 minutes at room temperature. The supernatant was carefully decanted, the pellet resuspended in MSC culture medium and plated onto gelatin-coated plates. After overnight incubation, the medium was changed every other day until the cells reached 80% confluency (4–5 days post-harvest).

### Protein, DNA, and RNA analyses

For western blot, cells were lysed in RIPA buffer with protease inhibitor cocktail (Roche). Protein lysates were quantified using a BCA quantification kit (Thermo Fisher Scientific), subjected to SDS-PAGE and electrotransferred to PVDF membranes (Millipore). Then the membranes were blocked with 5% milk, and incubated with primary antibodies and horseradish peroxidase (HRP)-conjugated secondary antibodies. The results of western blotting were obtained by ChemiDoc XRS system (Bio-Rad). For RT-qPCR, total RNA was extracted in TRIzol (Thermo Fisher Scientific) and genomic DNA was removed using a DNA-free kit (Thermo Fisher Scientific). cDNA was generated by the GoScript Reverse Transcription System (Promega). RT-qPCR was performed using the qPCR Mix (TOYOBO) in a CFX384 Real-Time system (Bio-Rad). For genomic PCR, a DNA extraction kit (TIANGEN) was used to extract genomic DNA and PCR was carried out with PrimeSTAR (TAKARA).

### Immunofluorescence microscopy

For immunofluorescence, cells seeded on coverslips (Thermo Fisher Scientific) were fixed in 4% paraformaldehyde (PFA) for 20 min, permeabilized with 0.4% Triton X-100 in PBS for 1 hour, and blocked with 10% donkey serum in PBS (Jackson ImmunoResearch) for 1 h at room temperature. Cells were then incubated with primary antibodies in the blocking buffer at 4 °C overnight, washed with PBS three times, incubated with secondary antibodies at room temperature for 1 h, washed again with PBS three times, and counterstained with Hoechst 33342 (Thermo Fisher Scientific). Leica SP5 confocal system was used for immunofluorescence microscopy. DNA texture image in one nucleus was measured with Coefficient of Variation (C.V) as previously described^9^. The degree of variation of all pixel value in one nucleus was measured by C.V of DNA texture image with ImageJ (C.V = Standard deviation/Mean).

### Transmission electron microscope (TEM)

One million cells of either WT or DR8^dex2^ hMSCs were harvested enzymatically by TrypLE (Thermo Fisher Scientific) and fixed with 4% PFA in PBS, pH 7.4, on ice for 2 h. Cells were subsequently dehydrated in a graded series of ethanol, infiltrated and embedded in Lowicryl resin HM20. Two hundred nanometer sections were obtained and imaged by a Spirit transmission electron microscope (FEI Company) operating at 100 kV.

### Mutagenesis of DGCR8

The vector expressing DGCR8 (mtDRBD) was generated by introducing multipoint mutations at coding sequences of two DGCR8 dsRBDs as previously described^25^. Point mutagenesis was carried out using a Fast Multisite Mutagenesis System (TRANSGEN BIOTECH) according to the manufacturer’s instructions.

### SA-β-gal staining assay

SA-β-gal staining of hMSCs was conducted as previously described^51^. Briefly, cells were fixed in 2% formaldehyde and 0.2% glutaraldehyde at room temperature for 4 min and stained with fresh staining solution at 37 °C overnight. SA-β-gal-positive cells were counted in randomly selected fields by Image J software.

### Clonal expansion assay

Cells were seeded at 2000 cells per well in 12-well plates and cultured for around 10 days. Cells were fixed in 4% PFA for 30 minutes and stained with 0.2% crystal violet for 1 h at room temperature. Cell numbers were counted in randomly selected fields and staining areas were measured by Image J software.

### AP staining analysis

hESCs were seeded at 2000 cells per well in 12-well plates and cultured for around 2 weeks. Cells were fixed and stained with Alkaline Phosphatase Stain Kit (Shanghai Yeasen Biotechnology Co. Ltd. China) according to the instructions. Stained areas were measured by Image J software (NIH).

### Lentivirus packaging

For packaging of lentiviruses, HEK293T cells were co-transfected with lentiviral vectors, psPAX2 (#12260, Addgene) as well as pMD2.G (#12259, Addgene). Viral particles were collected by ultracentrifugation at 19,400 g at 4 °C for 2.5 h.

### Co-immunoprecipitation

For exogenous Co-immunoprecipitation (Co-IP), HEK293T cells transfected with plasmid expressing Flag-DGCR8 were lysed in CHAPS lysis buffer (120 mM NaCl, 0.3% CHAPS, 1 mM EDTA, 40 mM HEPES, pH 7.5, and 1x complete protease inhibitor cocktail (Roche)). For endogenous Co-IP, hMSCs were lysed in CHAPS lysis buffer. HEK293T cells or hMSCs were lysed at 4 °C for 2 h, and then centrifuged at 12,000 g at 4 °C for 30 min. For endogenous Co-IP, lysates (1 mg protein) were pre-cleared with 20 μl of Protein A/G-PLUS Agarose beads (Santa Cruz) for 2–4 h, and then the supernatants were collected by centrifugation at 3 000 rpm at 4 °C for 3 min. The supernatants mixed with the indicated antibodies and beads were rotated overnight at 4 °C. The immunocomplexes were washed with CHAPS buffer three times and then eluted by boiling in 1 × SDS-loading buffer for 10 min.

### LC-MS/MS analysis

The elution proteins of immunoprecipitation were separated by 12% SDS-PAGE gel and visualized by Pierce™ Silver Stain (Thermo Fisher Scientific). Following decoloration and dehydration of the gels, the proteins were digested with sequencing-grade trypsin (Worthington). Subsequently, the peptides were extracted from gel pieces with 0.1% formic acid and 50% acetonitrile, dried in a vacuum centrifuge (ThermoFisher, San Jose, CA), dissolved in 0.1% formic acid (FA) and separated by a C18 reverse phase column (75 μm×20 cm, 3 μm). The column was then eluted with a linear gradient of 5-30% acetonitrile in 0.2% formic acid at the rate of 300 nL/min for 100 minutes. The mass spectra were acquired by nanoLC-Q EXACTIVE (ThermoFisher, San Jose, CA) equipped with a nano-ES ion source (Proxeon Biosystems, Denmark). Full scan spectra (from m/z 300–1600) were acquired in the Orbitrap analyzer with a resolution of 60,000 at 400 m/z after the accumulation of 1,000,000 ions. The five most-intense ions per scan were selected for collision-induced dissociation (CID) fragmentation in the linear ion trap after the accumulation of 3000 ions. The maximal filling times were set at 500 ms for the full scans and 150 ms for the MS/MS scans. The dynamic exclusion list was restricted to a maximum of 500 entries with a maximum retention period of 60 s and a relative mass window of 10 ppm.

All raw files were processed with the MaxQuant software (Version 1.3.0.5). The generated peak list files were searched with Thermo Proteome Discoverer (1.4.0.288) against the UniProt-proteome-human database (update-20160226). The search parameters were set as follows: enzyme was trypsin; up to two missed cleavages; alkylated cysteine as fixed modification; oxidation methionine as variable modifications. MS tolerance was 10 ppm while MS/MS tolerance was 0.02 Da. The required false discovery rate (FDR) was set to 1% at peptide and protein levels, and the minimum required peptide length was seven amino acids. At least one unique or razor peptide per protein group was required for protein identification.

### ChIP-qPCR and ChIP-seq

ChIP was performed according to a previous protocol with slight modifications^52^. Briefly, 1 × 10^6^ WT or DR8^dex2^ hMSCs were cross-linked in 1% vol/vol formaldehyde/PBS for 10 minutes at room temperature and then stopped by 125 mM Glycine for 5 min at room temperature. Samples were lysed on ice for 10 min. After sonicating by a Bioruptor® Plus sonication device (Diagenode), supernatants were incubated overnight at 4 °C with Protein G dynabeads (Thermo Fisher Scientific, 10004D) associated with 2.4 μg anti-DGCR8, H3K9me3 antibody or rabbit IgG. Subsequently, elution and reverse cross-link were performed at 68 °C for 2 h on a thermomixer. Then the DNA was isolated using phenol-chloroform-isoamylalcohol extraction and ethanol precipitation. The purified DNA was subjected to qPCR to evaluate DGCR8 or H3K9me3 occupation at satellite and LINE1 sites. The enriched fragments were constructed into libraries without the incorporation of spike-in controls via KAPA Hyper Prep Kits with PCR Library Amplification/Illumina series (KK8504) following the manufacturer’s instructions.

### RNA-seq and miRNA-seq

Total cellular RNA was extracted using TRIzol (Thermo Fisher Scientific) from 1 × 10^6^ cells per duplicate and genomic DNA was removed using a DNA-free kit (Thermo Fisher Scientific). Quality control and sequencing was done by Novogene Bioinformatics Technology Co. Ltd.

### CNV

DNeasy Blood & Tissue Kit (Qiagen) was applied to extract genomic DNA from 1 × 10^6^ cells per duplicate. Quality control and sequencing were done with standard protocols in Novogene Bioinformatics Technology Co. Ltd.

### RNA-seq data analysis

Paired-end raw reads were trimmed with Trim Galore (v0.4.5, http://www.bioinformatics.babraham.ac.uk/projects/trim_galore/) and cleaned reads were mapped to UCSC hg19 human genome or UCSC mm10 mouse genome using hisat2 (v2.0.4)^53^ with default parameters. Aligned reads were then annotated and counted by HTSeq (v0.6.1)^54^. Differentially expressed genes (DEGs) were calculated using DESeq2 with the cutoff as follows: Benjamini–Hochberg adjust *P* value (p.adjust) < 0.05 and absolute log_2_ (fold change) > 0.5. The Pearson correlation coefficient (R) of RNA-seq replicates was computed based on DESeq2 regularized logarithm (rlog) normalized read count^55^. GO enrichment analysis was performed using ClusterProfiler package (v3.6.0)^56^ at Benjamini–Hochberg adjust *P* value (p.adjust) < 0.05.

### ChIP-seq data analysis

The processing of ChIP-seq data followed standard pipeline without spike-in normalization^9,57–59^. Firstly, low-quality reads and adapters were trimmed by Trim Galore (v0.4.5). Then the cleaned reads were aligned to UCSC hg19 human reference genome using bowtie2 (v2.2.9)^60^ and PCR duplicates were removed by Samtools (v1.6)^61^. For H3K9me3 peak calling, MACS2 (v2.1.1)^62^ was used with default parameter.

The definition of “H3K9me3 mountains” was described previously^9^. Briefly, bam files of replicates were merged after alignment and called peaks by MACS2. Neighboring peaks were combined and “H3K9me3 mountains” were filtered at the cutoff 10 kb length. H3K9me3 mountains were then merged to union ones at the maximum distance between features 1 Mb.

Pearson correlation coefficient of replicates was computed based on DESeq2 regularized logarithm (rlog) normalized read count to evaluate the reproducibility.

For H3K9me3 ChIP-seq signal enrichment analysis at the regions of LADs and heterochromatin, we retained H3K9me3 signal enriched LAD and heterochromatin genomic regions. The average H3K9me3 signals were shown based on RPKM (Reads Per Kilobase Million) normalized read count on 10 bp bin size by deepTools2^63^. To investigate H3K9me3 signals of the lost “H3K9me3 mountains” regions in DR8^dex2^ hMSCs transduced with Luc, DR8-WT, or DR8-mtDRBD, heatmap was shown based on CPM (Counts Per Million) normalized read count.

For genomic elements enrichment analysis, ChIPseeker (v1.14.1)^64^ was used to annotate H3K9me3 ChIP-seq peaks. Genomic features were divided into four categories, “Promoter”, “Exon”, “Intron”, and “Intergenic”. For heterochromatin and LAD annotation of H3K9me3 ChIP-seq signal, the genomic locations of heterochromatin regions were obtained from Roadmap Epigenomics Mapping Consortium^65^. The genomic locations of LADs were downloaded from the UCSC hg19 goldenPath.

### microRNA-seq data analysis

The mirDeep2 pipeline was implemented for microRNA-seq data processing^66^. In brief, raw data was trimmed and then aligned to hg19 genome using bowtie. Known microRNAs were annotated by miRBase database^67^. Then the reads of each known microRNA were counted and the downstream analyzed by DESeq2. Normalized read count of each microRNA by DESeq2 size factor function was used for heatmap drawing.

### Copy number variation data analysis

Copy number variation (CNV) analysis was performed according to previously described pipeline^68^. Briefly, paired-end reads were trimmed with Trim Galore (v0.4.5) and then mapped to UCSC hg19 human genome using bowtie2 (v2.2.9)^60^. The CNVs were estimated and corrected by HMMcopy (v1.25.0) in each 0.5 Mb window^69^. The CNV plot was generated by R package circlize (v0.4.5)^70^.

### Teratoma formation assay

NOD/SCID mice were injected with 3 × 10^6^ hESCs suspended in a Matrigel/mTeSR (1:4) solution. Teratomas were collected at around 8 weeks after injection for further analysis.

### hMSC transplantation assay

In all, 1 × 10^6^ WT and DR8^dex2^ hMSCs pretransduced with lentiviruses expressing luciferase were injected into tibialis anterior (TA) muscle of male nude mice. 1/3/5 days after transplantation, mice were imaged by IVIS spectrum imaging system (XENOGEN, Caliper) for luciferase activity detection.

### ACLT-induced OA mouse model

An OA mouse model was generated as previously described^33^. Briefly, ACLT was performed on male C57BL/6 mice aged 8 weeks. For the ACLT surgery, after opening the joint capsule, the ACL was transected with microscissors under a surgical microscope. One week after surgery, lentiviruses expressing Luc, DR8-WT, or DR8-mtDRBD were injected into the articular cavity. At 7 weeks after lentivirus injection, the mice knee joints were examined by microCT analysis. The mice were then euthanized and the joints were collected for histological assessment of the medial tibial plateau joint and RNA-seq analysis. Total RNAs from three knee joints of each experimental group were mixed and then separated into three technical repeats for RNA-seq.

### Histology and immunohistochemistry

Collected mouse joints were fixed in 4% PFA for 2 days, decalcified in 5% methanoic acid for 7 days and embedded in paraffin. Sections (5 μm) were cut from the paraffin blocks, stained with Fast Green FCF (0.02%) and safranin O (0.1%), and quantified using the Osteoarthritis Research Society International (OARSI) scoring system.

Immunohistochemical staining was performed using DAB staining method. Briefly, the slides were deparaffinized and rehydrated. Antigen retrieval was performed using 1 mg/ml Trypsin in PBS (Sigma-Aldrich) digestion for 10 min. Slides were then incubated with endogenous peroxidase inhibitor (Beijing Zhongshan Jinqiao Biological Technology Co., Ltd.) at room temperature for 10 minutes to block endogenous peroxidase activity. Slides were washed and blocked with blocking solution (10% donkey serum in PBS) for 1 h at room temperature and then incubated with a primary antibody diluted in blocking solution overnight at 4 °C. Subsequently, slides were washed and incubated with secondary antibodies at room temperature for 30 min. After washed by PBS, the slides were visualized using DAB substrate (applied for less than 5 min until the desired color intensity was reached) and counterstained with hematoxylin.

### Statistical analysis

Results were presented as mean ± SEM. Two-tailed Student’s *t*-test was conducted using Graph-Pad Prism Software. *P* values < 0.05 were considered statistically significant (*).

### Ethics statement

All animal experiments were conducted according to animal protocols approved by the Chinese Academy of Science Institutional Animal Care and Use Committee. Mice were housed under a 12-hour light/dark cycle and supplied with food and water ad libitum. Mice were anaesthetized using isoflurane and euthanized with CO_2_ followed by cervical dislocation.

### Reporting summary

Further information on research design is available in the Nature Research Reporting Summary linked to this article.

## Supplementary information

Supplementary Information

Reporting Summary

Description of Additional Supplementary Files

Supplementary Data 1

Supplementary Data 2

Supplementary Data 3

Supplementary Data 4

Supplementary Data 5

Supplementary Data 6

Supplementary Data 7

Source Data

## Data Availability

Raw sequencing data and processed data of RNA-seq, microRNA-seq and ChIP-seq have been deposited in the NCBI Gene Expression Omnibus (GEO) under the accession number GSE113117, GSE121029, GSE116303, GSE113181 and GSE130206. The whole genome sequencing data have been deposited in the NCBI Sequence Read Archive under accession number PRJNA541163. The proteomics data have been deposited to the ProteomeXchange Consortium via the PRIDE partner repository with the dataset identifier PXD013856. The authors declare that all the data supporting the findings of this study are available within the article and its supplementary information files or from the corresponding author upon reasonable request. The source data underlying Figs. 1f, 1g, 2c-e, 2g, 2i-k, 3, 4c, 4f, 4g, 5, 6a, 6c-f, 7b-e, 8a, 9b-d and Supplementary Figs. 1h-k, 2b-d, 2f, 2h-i, 2k-m, 3f, 4d, 4f, 4g, 7a, 8a, 9c-j, and 10 are provided as a Source Data file. The uncropped blots and gels of Figs. 1b, 2b, 2f, 4a, 4b, 6b and Supplementary Figs. 1b, 1e, 1l-n, 2g, 2j, 3a, 3e, 4c, 4e, 7g, 7h, 9a and 9b are provided as Supplementary Figures 13-19.

## Code availability

High-throughput sequencing data analyses were performed using custom Python, Perl and R codes based on released softwares, as described in the Methods. All codes used in this study are available upon request from the corresponding authors. The software for data processing is available from the corresponding author upon request.

## References

[1] Villeponteau B (1997). The heterochromatin loss model of aging. Exp. Gerontol..

[2] Lombard DB (2005). DNA repair, genome stability, and aging. Cell.

[3] Lopez-Otin C, Blasco MA, Partridge L, Serrano M, Kroemer G (2013). The hallmarks of aging. Cell.

[4] Ren R, Ocampo A, Liu GH, Izpisua Belmonte JC (2017). Regulation of stem cell aging by metabolism and epigenetics. Cell Metab..

[5] Liang R, Ghaffari S (2014). Stem cells, redox signaling, and stem cell aging. Antioxid. redox Signal..

[6] Uccelli A, Moretta L, Pistoia V (2008). Mesenchymal stem cells in health and disease. Nat. Rev. Immunol..

[7] Kubben N (2016). Repression of the antioxidant NRF2 pathway in premature. Aging Cell.

[8] Kudlow BA, Kennedy BK, Monnat RJ (2007). Werner and Hutchinson-Gilford progeria syndromes: mechanistic basis of human progeroid diseases. Nat. Rev. Mol. cell Biol..

[9] Zhang W (2015). Aging stem cells. A Werner syndrome stem cell model unveils heterochromatin alterations as a driver of human aging. Science.

[10] Ren R (2017). Visualization of aging-associated chromatin alterations with an engineered TALE system. Cell Res..

[11] Oberdoerffer P, Sinclair DA (2007). The role of nuclear architecture in genomic instability and ageing. Nat. Rev. Mol. cell Biol..

[12] Shumaker DK (2006). Mutant nuclear lamin A leads to progressive alterations of epigenetic control in premature aging. Proc. Natl Acad. Sci. USA.

[13] Wu Zeming, Zhang Weiqi, Song Moshi, Wang Wei, Wei Gang, Li Wei, Lei Jinghui, Huang Yu, Sang Yanmei, Chan Piu, Chen Chang, Qu Jing, Suzuki Keiichiro, Belmonte Juan Carlos Izpisua, Liu Guang-Hui (2018). Differential stem cell aging kinetics in Hutchinson-Gilford progeria syndrome and Werner syndrome. Protein & Cell.

[14] Larson K (2012). Heterochromatin formation promotes longevity and represses ribosomal RNA synthesis. PLoS Genet..

[15] Li Y (2016). Vitamin C alleviates aging defects in a stem cell model for Werner syndrome. Protein Cell.

[16] Gregory RI (2004). The microprocessor complex mediates the genesis of microRNAs. Nature.

[17] Nguyen TA (2015). Functional anatomy of the human microprocessor. Cell.

[18] Roth BM, Ishimaru D, Hennig M (2013). The core microprocessor component digeorge syndrome critical region 8 (DGCR8) is a nonspecific RNA-binding protein. J. Biol. Chem..

[19] Wang Y, Medvid R, Melton C, Jaenisch R, Blelloch R (2007). DGCR8 is essential for microRNA biogenesis and silencing of embryonic stem cell self-renewal. Nat. Genet..

[20] Brandl A (2016). The microprocessor component, DGCR8, is essential for early B-cell development in mice. Eur. J. Immunol..

[21] Calses PC (2017). DGCR8 mediates repair of UV-Induced DNA damage independently of RNA processing. Cell Rep..

[22] Ding Q (2013). Enhanced efficiency of human pluripotent stem cell genome editing through replacing TALENs with CRISPRs. cell stem cell.

[23] Mali P (2013). RNA-guided human genome engineering via Cas9. Science.

[24] Yang J (2017). Genetic enhancement in cultured human adult stem cells conferred by a single nucleotide recoding. Cell Res.

[25] Yeom KH, Lee Y, Han J, Suh MR, Kim VN (2006). Characterization of DGCR8/Pasha, the essential cofactor for Drosha in primary miRNA processing. Nucleic Acids Res..

[26] Bulut-Karslioglu A (2014). Suv39h-dependent H3K9me3 marks intact retrotransposons and silences LINE elements in mouse embryonic stem cells. Mol. cell.

[27] Peters AH (2001). Loss of the Suv39h histone methyltransferases impairs mammalian heterochromatin and genome stability. Cell.

[28] Wang D (2013). Methylation of SUV39H1 by SET7/9 results in heterochromatin relaxation and genome instability. Proc. Natl Acad. Sci. USA.

[29] Van Meter M (2014). SIRT6 represses LINE1 retrotransposons by ribosylating KAP1 but this repression fails with stress and age. Nat. Commun..

[30] Ianni A, Hoelper S, Krueger M, Braun T, Bober E (2017). Sirt7 stabilizes rDNA heterochromatin through recruitment of DNMT1 and Sirt1. Biochem. Biophys. Res. Commun..

[31] Siljak-Yakovlev S (2017). Evolutionary implications of heterochromatin and rDNA in chromosome number and genome size changes during dysploidy: A case study in Reichardia genus. PloS one.

[32] Jeon OH, David N, Campisi J, Elisseeff JH (2018). Senescent cells and osteoarthritis: a painful connection. J. Clin. Investig..

[33] Jeon OH (2017). Local clearance of senescent cells attenuates the development of post-traumatic osteoarthritis and creates a pro-regenerative environment. Nat. Med..

[34] Chen Z (2012). DiGeorge syndrome critical region 8 (DGCR8) protein-mediated microRNA biogenesis is essential for vascular smooth muscle cell development in mice. J. Biol. Chem..

[35] Wang Y (2008). Embryonic stem cell-specific microRNAs regulate the G1-S transition and promote rapid proliferation. Nat. Genet..

[36] Yi R (2009). DGCR8-dependent microRNA biogenesis is essential for skin development. Proc. Natl Acad. Sci. USA.

[37] Cheng TL (2014). MeCP2 suppresses nuclear microRNA processing and dendritic growth by regulating the DGCR8/Drosha complex. Dev. cell.

[38] Marinaro F (2017). MicroRNA-independent functions of DGCR8 are essential for neocortical development and TBR1 expression. EMBO Rep..

[39] Cirera-Salinas D (2017). Noncanonical function of DGCR8 controls mESC exit from pluripotency. J. cell Biol..

[40] Peng JC, Karpen GH (2007). H3K9 methylation and RNA interference regulate nucleolar organization and repeated DNA stability. Nat. cell Biol..

[41] Liu Z, Skamagki M, Kim K, Zhao R (2015). Canonical microRNA activity facilitates but may be dispensable for transcription factor-mediated reprogramming. Stem Cell Rep..

[42] Geng Lingling, Liu Zunpeng, Zhang Weiqi, Li Wei, Wu Zeming, Wang Wei, Ren Ruotong, Su Yao, Wang Peichang, Sun Liang, Ju Zhenyu, Chan Piu, Song Moshi, Qu Jing, Liu Guang-Hui (2018). Chemical screen identifies a geroprotective role of quercetin in premature aging. Protein & Cell.

[43] McGonagle D, Baboolal TG, Jones E (2017). Native joint-resident mesenchymal stem cells for cartilage repair in osteoarthritis. Nat. Rev. Rheumatol..

[44] Ren X (2019). Maintenance of nucleolar homeostasis by CBX4 alleviates senescence and osteoarthritis. Cell Rep..

[45] Fu L (2019). Up-regulation of FOXD1 by YAP alleviates senescence and osteoarthritis. PLoS Biol..

[46] Suzuki K (2016). In vivo genome editing via CRISPR/Cas9 mediated homology-independent targeted integration. Nature.

[47] Wang S (2018). ATF6 safeguards organelle homeostasis and cellular aging in human mesenchymal stem cells. Cell Discov..

[48] Sanjana NE, Shalem O, Zhang F (2014). Improved vectors and genome-wide libraries for CRISPR screening. Nat. methods.

[49] Shalem O (2014). Genome-scale CRISPR-Cas9 knockout screening in human cells. Science.

[50] Pan H (2016). SIRT6 safeguards human mesenchymal stem cells from oxidative stress by coactivating NRF2. Cell Res..

[51] Debacq-Chainiaux F, Erusalimsky JD, Campisi J, Toussaint O (2009). Protocols to detect senescence-associated beta-galactosidase (SA-betagal) activity, a biomarker of senescent cells in culture and in vivo. Nat. Protoc..

[52] Dahl JA, Collas P (2008). A rapid micro chromatin immunoprecipitation assay (microChIP). Nat. Protoc..

[53] Kim D, Langmead B, Salzberg SL (2015). HISAT: a fast spliced aligner with low memory requirements. Nat. methods.

[54] Anders S, Pyl PT, Huber W (2015). HTSeq—a Python framework to work with high-throughput sequencing data. Bioinformatics.

[55] Love MI, Huber W, Anders S (2014). Moderated estimation of fold change and dispersion for RNA-seq data with DESeq2. Genome Biol..

[56] Yu G, Wang LG, Han Y, He QY (2012). clusterProfiler: an R package for comparing biological themes among gene clusters. Omics: a J. Integr. Biol..

[57] Hansen A S, Pustova I, Cattoglio C, Tjian R, Darzacq X. (2017). CTCF and cohesin regulate chromatin loop stability with distinct dynamics. eLife.

[58] Ramirez F (2018). High-resolution TADs reveal DNA sequences underlying genome organization in flies. Nat. Commun..

[59] Hummel B (2017). The evolutionary capacitor HSP90 buffers the regulatory effects of mammalian endogenous retroviruses. Nat. Struct. Mol. Biol..

[60] Langmead B, Salzberg SL (2012). Fast gapped-read alignment with Bowtie 2. Nat. methods.

[61] Li H (2009). The sequence alignment/Map format and SAMtools. Bioinformatics.

[62] Zhang Y (2008). Model-based analysis of ChIP-Seq (MACS). Genome Biol..

[63] Ramirez F (2016). deepTools2: a next generation web server for deep-sequencing data analysis. Nucleic acids Res..

[64] Yu G, Wang LG, He QY (2015). ChIPseeker: an R/Bioconductor package for ChIP peak annotation, comparison and visualization. Bioinformatics.

[65] Bernstein BE (2010). The NIH Roadmap Epigenomics Mapping Consortium. Nat. Biotechnol..

[66] Friedlander MR, Mackowiak SD, Li N, Chen W, Rajewsky N (2012). miRDeep2 accurately identifies known and hundreds of novel microRNA genes in seven animal clades. Nucleic acids Res..

[67] Kozomara A, Griffiths-Jones S (2014). miRBase: annotating high confidence microRNAs using deep sequencing data. Nucleic acids Res..

[68] Yan Pengze, Li Qingqing, Wang Lixia, Lu Ping, Suzuki Keiichiro, Liu Zunpeng, Lei Jinghui, Li Wei, He Xiaojuan, Wang Si, Ding Jianjian, Chan Piu, Zhang Weiqi, Song Moshi, Izpisua Belmonte Juan Carlos, Qu Jing, Tang Fuchou, Liu Guang-Hui (2019). FOXO3-Engineered Human ESC-Derived Vascular Cells Promote Vascular Protection and Regeneration. Cell Stem Cell.

[69] Ha G (2012). Integrative analysis of genome-wide loss of heterozygosity and monoallelic expression at nucleotide resolution reveals disrupted pathways in triple-negative breast cancer. Genome Res..

[70] Gu Z, Gu L, Eils R, Schlesner M, Brors B (2014). circlize Implements and enhances circular visualization in R. Bioinformatics.

