# Supplementary material for: Stabilizing heterochromatin by DGCR8 alleviates senescence and osteoarthritis
Source: Nat Commun. 2019 Jul 26;10:3329. doi: 10.1038/s41467-019-10831-8 (PMC6659673; doi:10.1038/s41467-019-10831-8)
Supplement: Supplementary file 1 — Supplementary Information [file 41467_2019_10831_MOESM1_ESM.pdf]

Supplementary Information

Deng et al.: Stabilizing Heterochromatin by DGCR8 Alleviates Senescence and Osteoarthritis

Supplementary Figure 1

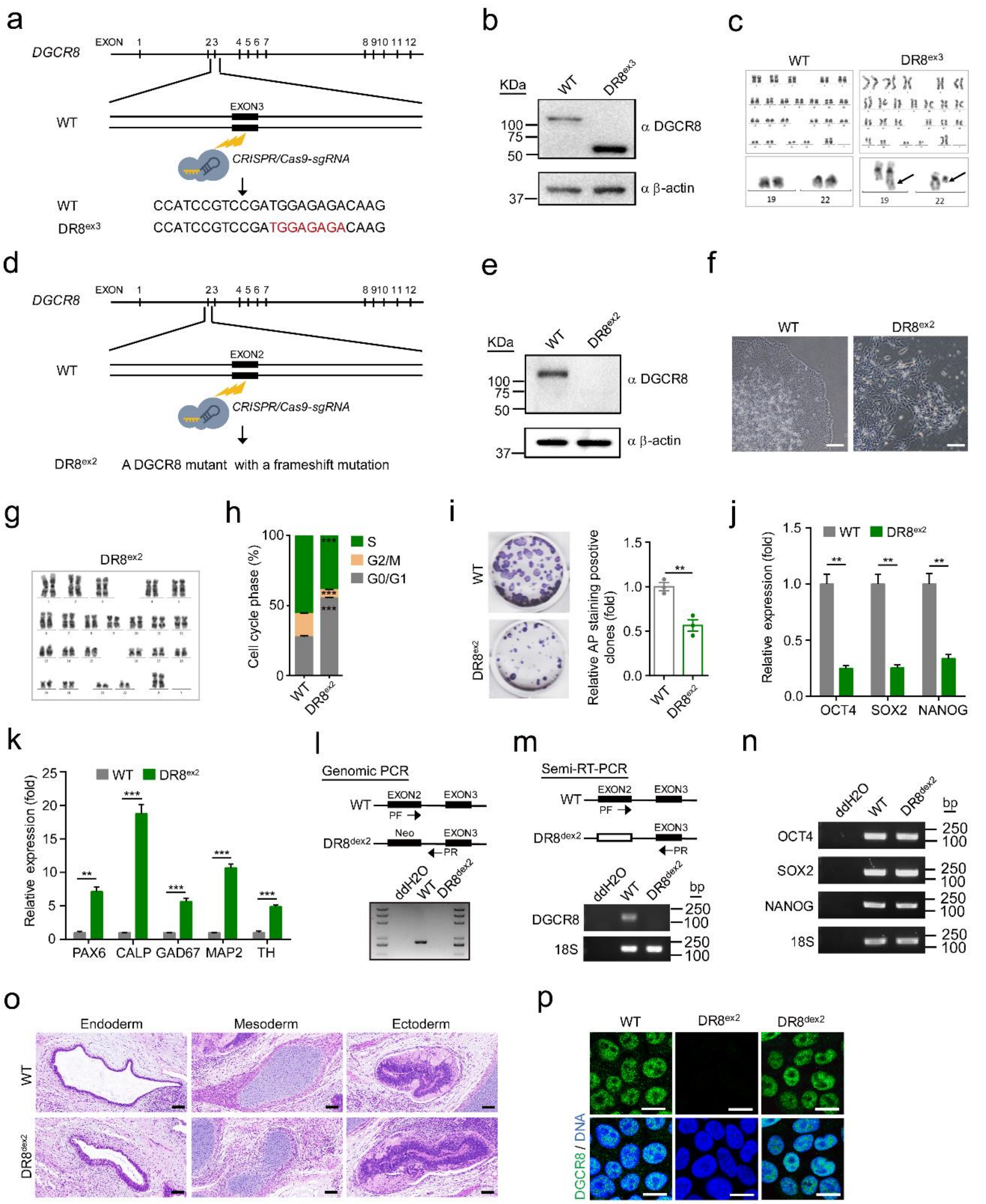

Supplementary Figure 1. Generation of DGCR8-defective hESCs. (**a**) Schematic demonstration of DGCR8 gene editing (exon 3) using CRISPR/Cas9-mediated NHEJ in hESCs. The sequencing result was shown at the bottom. The deleted nucleotides were marked in red. (**b**) Western blotting analysis of DGCR8 in wild-type (WT) and DR8^{ex3} hESCs. β-actin was used as the loading control. (**c**) G-banded karyotyping analysis of WT and DR8^{ex3} hESCs. Translocation between chromosome 19 and 22 was observed in DR8^{ex3} hESCs. (**d**) Schematic demonstration of DGCR8 gene editing (exon 2) using CRISPR/Cas9-mediated NHEJ in hESCs. (**e**) Western blotting analysis of DGCR8 in WT and DR8^{ex2} hESCs. β-actin was used as the loading control. (**f**) Morphology of WT and DR8^{ex2} hESCs. Scale bar, 100 µm. (**g**) G-banded karyotyping analysis of DR8^{ex2} hESCs. (**h**) Cell cycle analysis of WT and DR8^{ex2} hESCs. Data were presented as mean ± SEM. n=3 wells per cell type, ***$p < 0.001$. (**i**) AP staining analysis of WT and DR8^{ex2} hESCs. Data were presented as mean ± SEM. n=3 wells per cell type, **$p < 0.01$. (**j**) RT-qPCR analysis of the pluripotency markers in WT and DR8^{ex2} hESCs. Data were presented as mean ± SEM. n=3 wells per condition, **$p < 0.01$. (**k**) RT-qPCR analysis of differentiation markers in WT and DR8^{ex2} hESCs. Data were presented as mean ± SEM. n=3 wells per condition, **$p < 0.01$, ***$p < 0.001$. (**l**) Genotyping of WT and DR8^{dex2} hESCs with primers spanning the region from exon 2 to the junction region between exon 2 and exon 3. (**m**) RT-PCR analysis for gene targeting identification with mRNA of WT and DR8^{dex2} hESCs. 18S rRNA was used as the control. (**n**) RT-PCR analysis of the pluripotency markers including OCT4, SOX2, and NANOG in WT and DR8^{dex2} hESCs. 18S rRNA was used as the control. (**o**) HE staining analysis of typical structures of three germ layers in teratomas derived from either WT or DR8^{dex2} hESCs. Scale bar, 100 µm. (**p**) Immunostaining of DGCR8 in WT, DR8^{ex2}, and DR8^{dex2} hESCs. Scale bar, 5 µm. Statistical significances were assessed by a two-tailed unpaired Student's t test.

Supplementary Figure 2

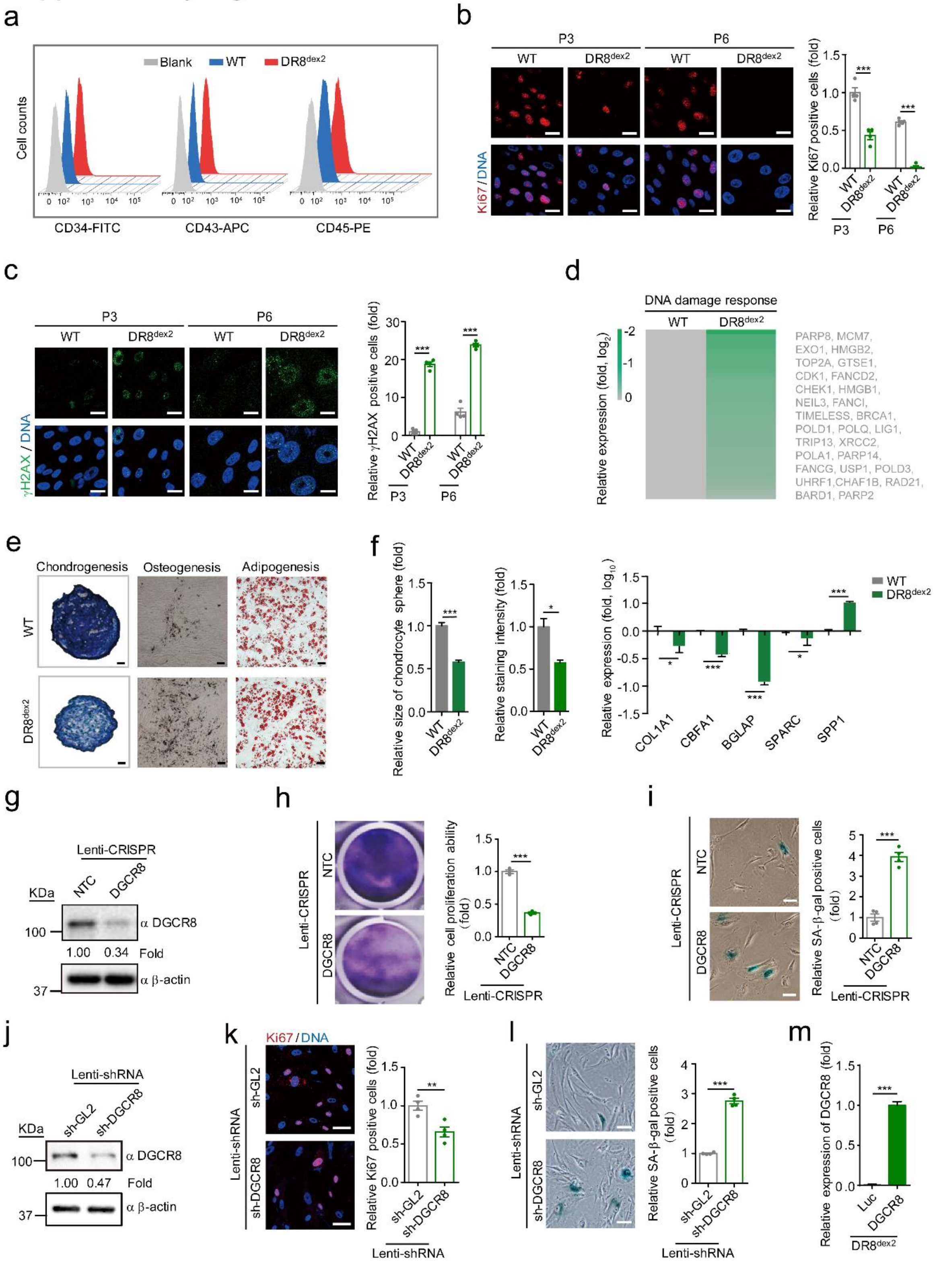

Supplementary Figure 2. Characterization of DR8^{dex2} hMSCs. (**a**) FACS analysis of WT and DR8^{dex2} hMSCs with MSC negative surface markers. (**b**) Immunostaining of Ki67 in WT and DR8^{dex2} hMSCs. Data were presented as mean ± SEM. n=4 images per cell type, ***$p < 0.001$. Scale bar, 25 µm. (**c**) Immunostaining of γH2AX in WT and DR8^{dex2} hMSCs. Data were presented as mean ± SEM. n=4 images per cell type, ***$p < 0.001$. Scale bar, 25 µm. (**d**) Heatmap showing expression levels of the DNA damage response-associated genes in DR8^{dex2} hMSCs compared with WT hMSCs. (**e**) Characterization of chondrogenesis, osteogenesis, and adipogenesis potentials in WT and DR8^{dex2} hMSCs, respectively. Scale bar, 20 µm. (**f**) Left and middle, measurement of the size and staining intensity of chondrocyte spheres derived from WT and DR8^{dex2} hMSCs. Right, RT-qPCR analysis of osteoblast-specific markers in the osteoblast derivatives differentiated from WT and DR8^{dex2} hMSCs. Data were presented as mean ± SEM. n=3 wells per condition, *$p < 0.05$, ***$p < 0.001$. (**g**) Western blotting analysis of DGCR8 in hMSCs transduced with lentivirus-CRISPRv2 targeting exon 5 of *DGCR8*. NTC means Non-Targeting Control. (**h**) Clonal expansion analysis of NTC and lentivirus-CRISPRv2 mediated DGCR8-knockout hMSCs. Data were presented as mean ± SEM. n=3 wells per cell type, ***$p < 0.001$. (**i**) SA-β-gal staining analysis of NTC and lentivirus-CRISPRv2 mediated DGCR8-targeted hMSCs. Data were presented as mean ± SEM. n=4 images per condition, ***$p < 0.001$. Scale bar, 20 µm. (**j**) Western blotting analysis of DGCR8 in hMSCs transduced with lentiviruses expressing shRNA-DGCR8 or shRNA-GL2. (**k**) Immunostaining of Ki67 in control and DGCR8-knockdown hMSCs. Data were presented as mean ± SEM. n=4 images per condition, ***$p < 0.001$. Scale bar, 50 µm. (**l**) SA-β-gal staining in control and DGCR8-knockdown hMSCs. Data were presented as mean ± SEM. n=4 images per condition, ***$p < 0.001$. Scale bar, 20 µm. (**m**) RT-qPCR detection of DGCR8 in DR8^{dex2} hMSCs transduced with lentiviruses expressing Luc or DGCR8. Data were presented as mean ± SEM. n=3 wells per condition, ***$p < 0.001$. Statistical significances were assessed by a two-tailed unpaired Student's t test.

Supplementary Figure 3

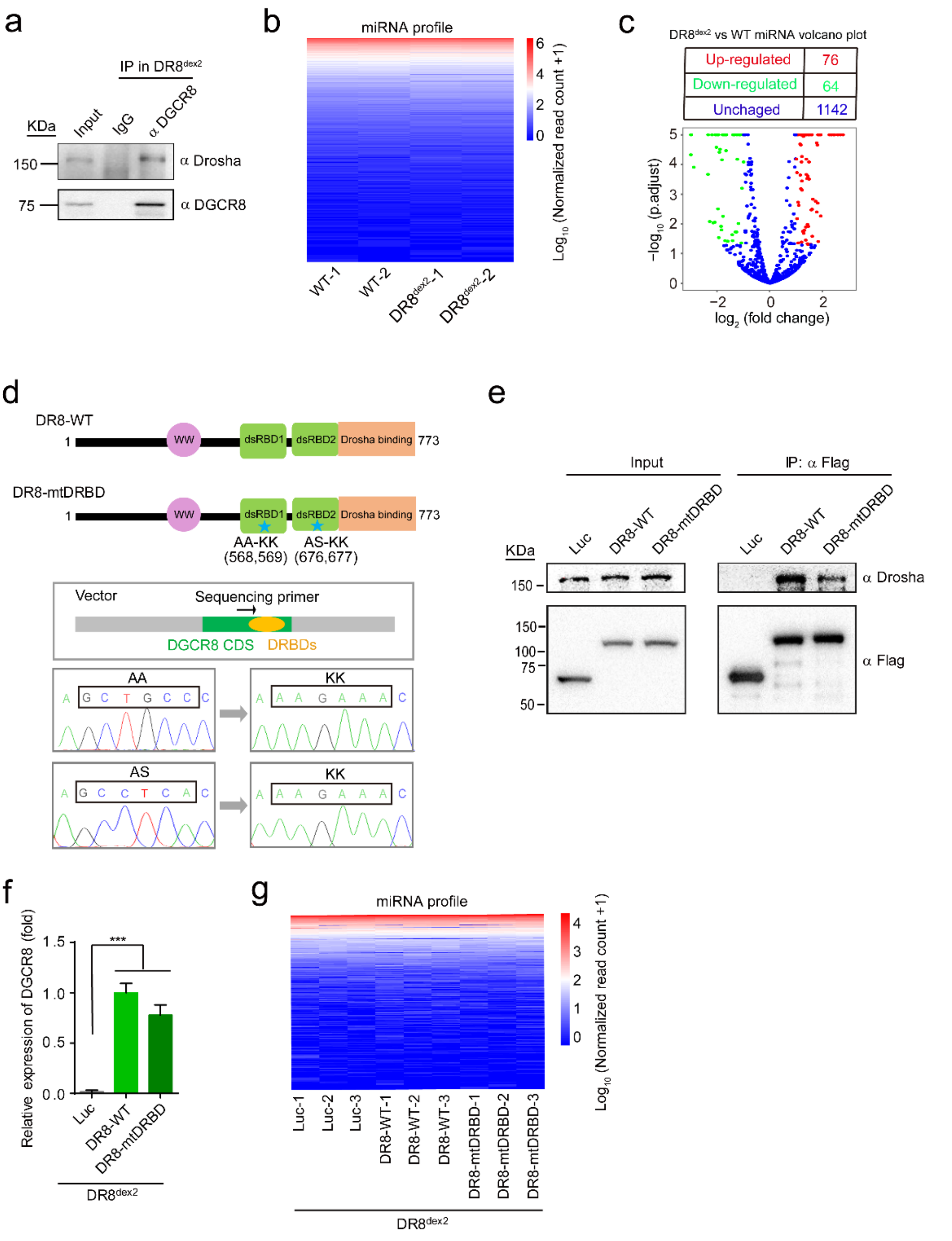

Supplementary Figure 3. miRNA processing function is unaffected in DR8^{dex2} hMSCs. (**a**) Co-immunoprecipitation analysis of Drosha protein with endogenous truncated DGCR8 proteins in DR8^{dex2} hMSCs. (**b**) Heatmap showing normalized expression levels of miRNAs in WT and DR8^{dex2} hMSCs. The expression levels of miRNAs in WT and DR8^{dex2} hMSCs were comparable. (**c**) Volcano plot showing unchanged and differentially expressed miRNAs between WT and DR8^{dex2} hMSCs. The differentially expressed miRNAs were listed in Supplementary Data 5. (**d**) The sketch map of DR8-mtDRBD and verification of the mutated bases. (**e**) Co-immunoprecipitation analysis of Drosha with exogenous Flag-tagged DR8-WT or DR8-mtDRBD protein in HEK293T cells. DR8-mtDRBD still keeps the ability to interact with Drosha. (**f**) RT-qPCR detection of DGCR8 expression levels in DR8^{dex2} hMSCs transduced with lentiviruses expressing Luc, DR8-WT or DR8-mtDRBD. Data were presented as mean ± SEM. n=3 wells per condition, ***$p < 0.001$. (**g**) Heatmap showing the miRNA profile of DR8^{dex2} hMSCs treated with lentiviruses expressing Luc, DR8-WT, or DR8-mtDRBD. Statistical significances were assessed by a two-tailed unpaired Student's t test.

Supplementary Figure 4

a

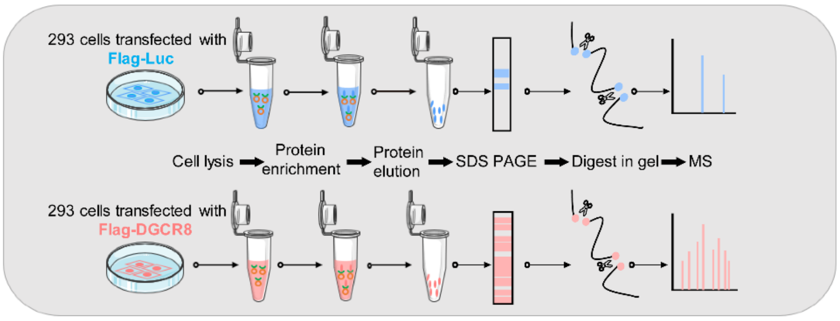

b

Gene name	Function	Coverage (%)	Unique peptides	Peptide sequence
Drosha	miRNA processing	1.17	1	1. TAIVQNQHLAMLAK
KAP1	Heterochromatin matainence	31.86	12	1. STAPSAAASASASAAASSPAGGGAEALELLEHcGVcR<br>2. LTEDKADVQSIIGLQR<br>3. LDLDLTADSQPPVFK<br>4. VLLALFcHEPcRPLHQLATDSTFSLDQPGGTLDLTLIR<br>5. DcQLNAHKDHQYQFLEDAVR<br>6. HEPLVLFcESCDTLTcR<br>7. IVAERPGTNSTGPAPMAPPR<br>8. QGSGSSQPMEVQEGYGFGSGDDPYSSAEPHVSGVKR<br>9. DIVENYFMR<br>10. TVYcNVHKHEPLVLFcEScDTLTcR<br>11. LLPcLHSAcSAcLGPAAPAAANSSGDGGAAGDGTVVDcPVcK<br>12. KLLASLVK

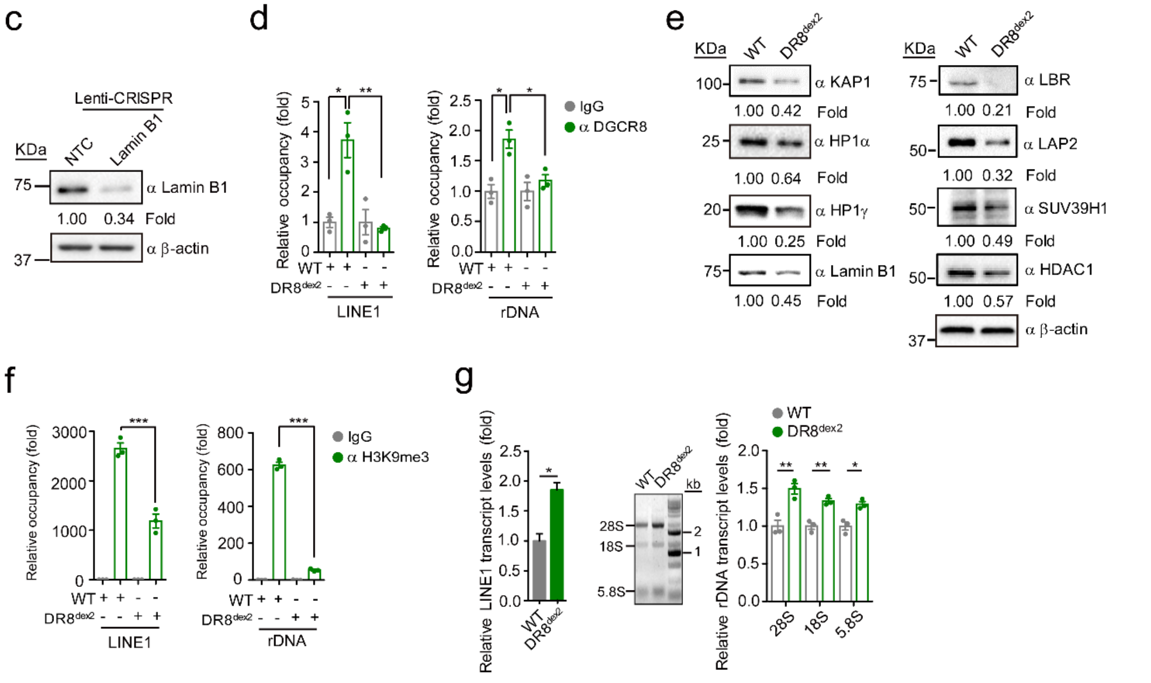

Supplementary Figure 4. DGCR8 is involved in heterochromatin stabilization. **(a)** The flow chart of mass spectrometry for identifying interacting proteins of DGCR8. Luc was used as control. The interacting proteins of DGCR8 identified by mass spectrometry were listed in Supplementary Data 6. **(b)** Interaction between Drosha and KAP1 and DGCR8 identified by mass spectrometry. Unique peptides of each interacting proteins were listed in the table. **(c)** Western blotting analysis of Lamin B1 for the validation of antibody specificity in hMSCs transduced with lentivirus-CRISPRv2 targeting *LMNB1*. β-actin was used as the loading control. NTC means non-targeting control. **(d)** Enrichment of DGCR8 within the region of LINE1 and rDNA as measured by ChIP-qPCR. Data were presented as mean ± SEM. n=3 wells per condition, *$p < 0.05$, **$p < 0.01$. **(e)** Western blotting analysis of heterochromatin-related proteins in hMSCs. β-actin was used as the loading control. **(f)** Enrichment of H3K9me3 within the region of LINE1 and rDNA as measured by ChIP-qPCR. Data were presented as mean ± SEM. n=3 wells per condition, ***$p < 0.001$. **(g)** Left, quantitative RT-PCR analysis of LINE1 transcripts in WT and DR8^{dex2} hMSCs. Middle and right, gel electrophoresis and statistical analysis of rDNA transcripts in WT and DR8^{dex2} hMSCs. Total RNA was extracted from equal number of WT and DR8^{dex2} hMSCs. Data were presented as mean ± SEM. n=3 wells per condition, *$p < 0.05$, **$p < 0.01$. Statistical significances were assessed by a two-tailed unpaired Student's t test.

Supplementary Figure 5

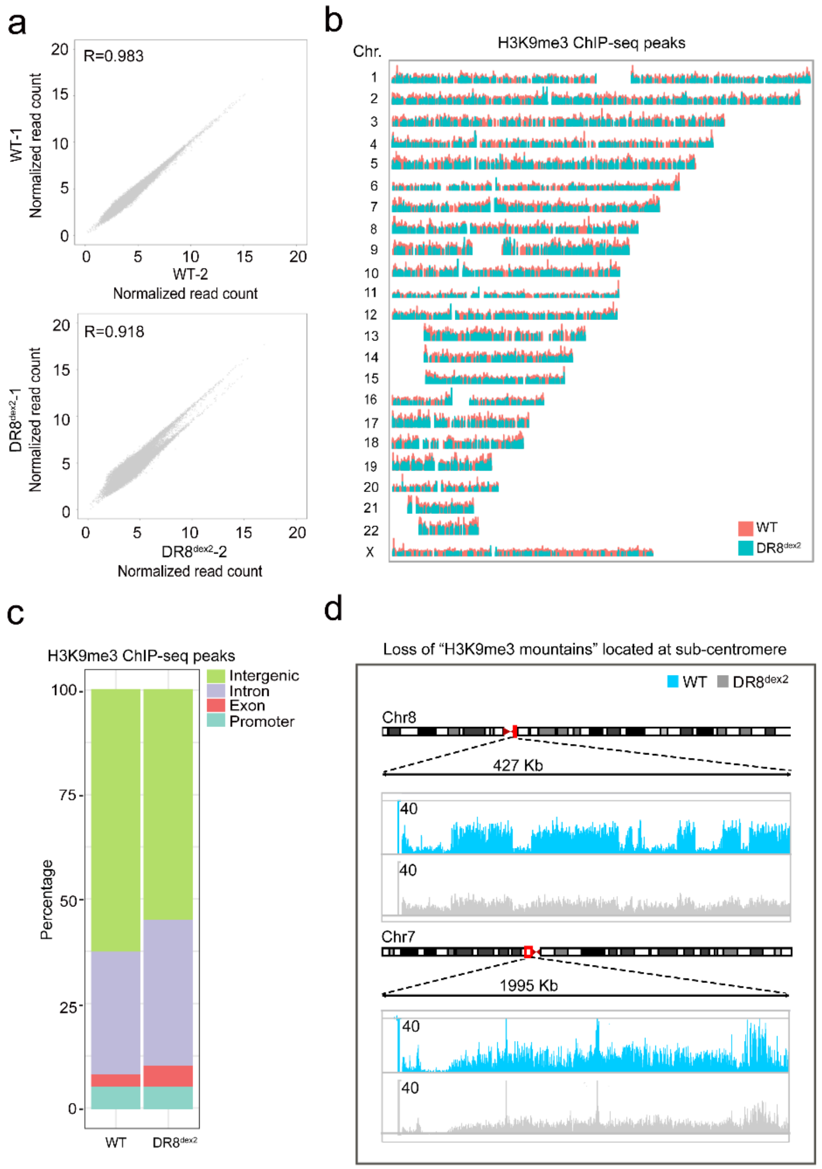

Supplementary Figure 5. H3K9me3 ChIP-seq in WT and DR8^{dex2} hMSCs. (**a**) Scatter plot showing the correlation between ChIP-seq replicates of WT or DR8^{dex2} hMSCs. Pearson correlation coefficient (R) is presented. (**b**) ChIP-seq profile showing the distribution of H3K9me3 peaks over all chromosomes of WT and DR8^{dex2} hMSCs. (**c**) Bar plot showing genomic distribution of H3K9me3 peaks in gene bodies and intergenic regions. (**d**) Representative images showing two “H3K9me3 mountains” on chromosome 7 and chromosome 8 in the sub-centromere regions in WT and DR8^{dex2} hMSCs. Red rectangles denote the position of the presented sub-centromere regions.

Supplementary Figure 6

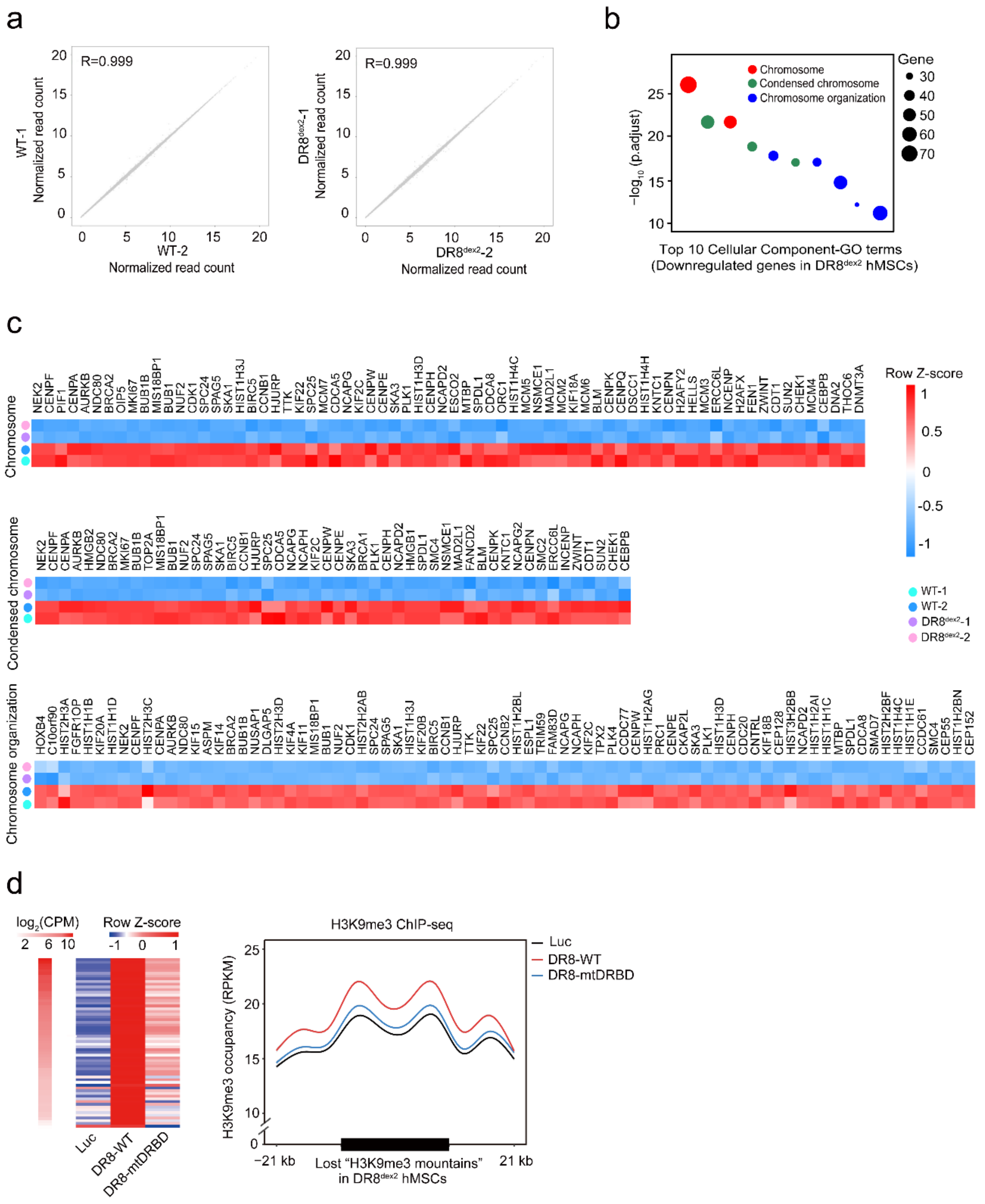

Supplementary Figure 6. RNA-seq in WT and DR8^{dex2} hMSCs. (**a**) Scatter plot showing the correlation between RNA-seq replicates of WT or DR8^{dex2} hMSCs. Pearson correlation coefficient (R) is presented. (**b**) Dot plot showing the top 10 enriched cellular component GO terms in the downregulated genes of DR8^{dex2} hMSCs. (**c**) Heatmap showing the Z-score normalized expression levels of genes associated with chromosome (top), condensed chromosome (middle) and chromosome organization (bottom) in DR8^{dex2} hMSCs. (**d**) Left, heatmap showing H3K9me3 signals at "H3K9me3 mountains"-lost regions in DR8^{dex2} hMSCs transduced with Luc, DR8-WT, or DR8-mtDRBD. Right, enrichment of H3K9me3 ChIP-seq signals ranging from 21 kb upstream to 21 kb downstream of "H3K9me3 mountains"-lost regions in DR8^{dex2} hMSCs transduced with Luc, DR8-WT, or DR8-mtDRBD.

Supplementary Figure 7

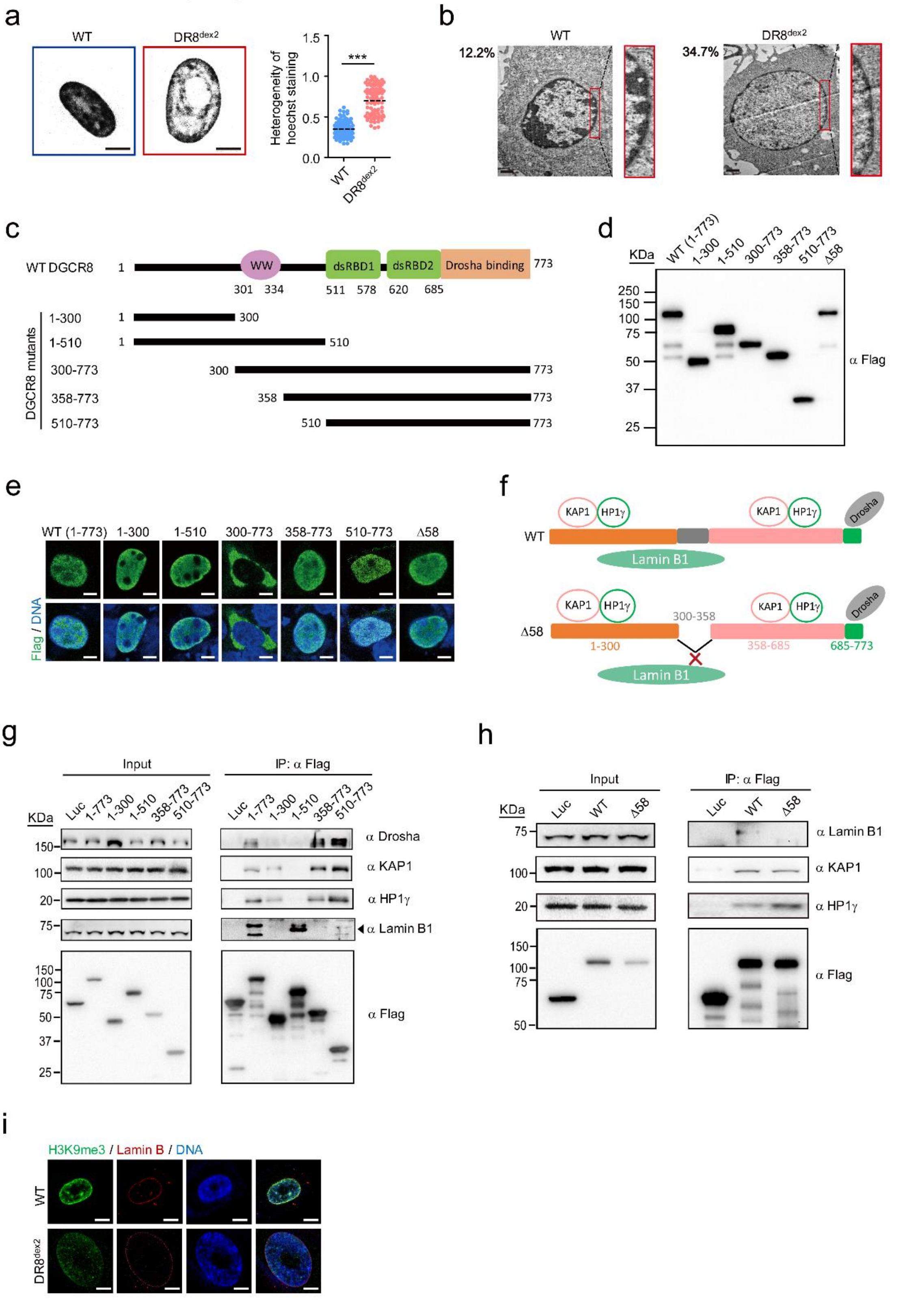

Supplementary Figure 7. DGCR8 maintains heterochromatin structure via the interaction with heterochromatin components. (**a**) Left: Chromatin distribution pattern in WT and DR8^{dex2} hMSCs indicated by nucleus staining with Hoechst 33342. Right: CV value of nuclear Hoechst staining intensity used to evaluate the heterogeneity (pixel-to-pixel variation) of the chromatin status. Data were presented as mean ± SEM. n=90 cells per cell type, ***p < 0.001. Scale bar, 25 μm. (**b**) The electron microscope (EM) analysis of the heterochromatin architecture at peripheral nucleus in P3 WT and DR8^{dex2} hMSCs. The percentages of cells with heterochromatin loss at nuclear periphery were presented at the upper left corner. Scale bar, 1 μm. (**c**) Schematic diagram of different DGCR8 truncations. (**d**) Flag immunoblotting of DGCR8 truncations. (**e**) Flag immunostaining of DGCR8 truncations. Except a.a. 300-773, other truncations together with WT DGCR8 (a.a. 1-773) localize in the nuclear. Δ58, 1-773 lack 300-358 amino acids. Scale bar, 5 μm. (**f**) Upper: Schematic diagram of WT DGCR8 protein (a.a. 1-773) and the possible domains interacting with KAP1, Lamin B1, and HP1γ according to the results of co-immunoprecipitation in Supplementary Fig. 7g. Lower: Schematic diagram of Δ58 mutant lacking the amino acids 300-358 which is the region required for the interaction between DGCR8 and Lamin B1. (**g**) Co-immunoprecipitation of Drosha, KAP1, HP1γ, and Lamin B1 protein with exogenous full-length and truncated DGCR8 proteins in HEK 293T cells. (**h**) Co-immunoprecipitation of Lamin B1, KAP1, and HP1γ protein with exogenous full-length DGCR8 and Δ58 mutant in HEK 293T cells. Δ58 mutant loses the binding ability to Lamin B1, but still keeps the binding ability to KAP1 and HP1γ. (**i**) Immunostaining of H3K9me3 and Lamin B in WT and DR8^{dex2} hMSCs. Scale bar, 10 μm. Statistical significances were assessed by a two-tailed unpaired Student's t test.

Supplementary Figure 8

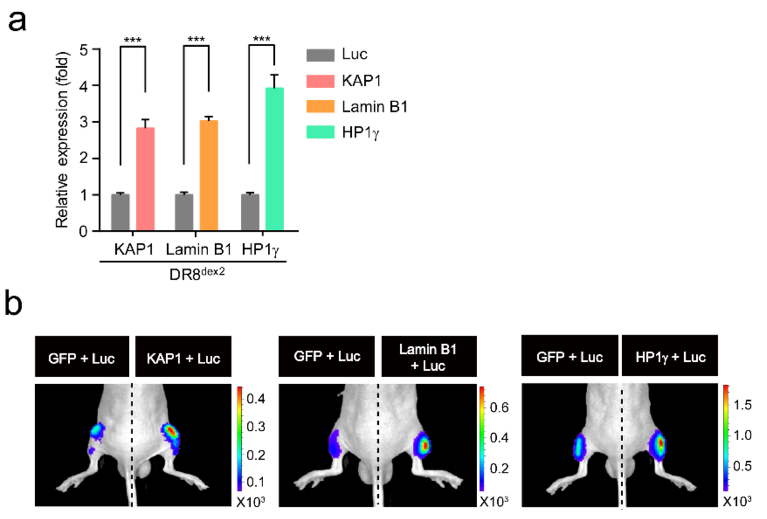

Supplementary Figure 8. Expression of DGCR8-interacting proteins rescues the senescence phenotypes of DR8^{dex2} hMSCs. (**a**) RT-qPCR detection of KAP1, Lamin B1, or HP1γ in DR8^{dex2} hMSCs transduced with lentiviruses expressing Luc, KAP1, Lamin B1, or HP1γ. Data were presented as mean ± SEM. n=3 wells per condition, ***$p < 0.001$. (**b**) Photon flux from tibialis anterior (TA) muscles of nude mice transplanted with DR8^{dex2} hMSCs with co-infection of lentiviral vectors expressing GFP or KAP1 (left) / Lamin B1 (middle) / HP1γ (right) and lentiviral vector expressing luciferase. Luciferase activity in the TA muscles was detected by an in vivo imaging system (IVIS) 4 days after implantation, demonstrating slower attrition of DR8^{dex2} hMSCs transduced with KAP1, Lamin B1, or HP1γ. Statistical significances were assessed by a two-tailed unpaired Student's t test.

Supplementary Figure 9

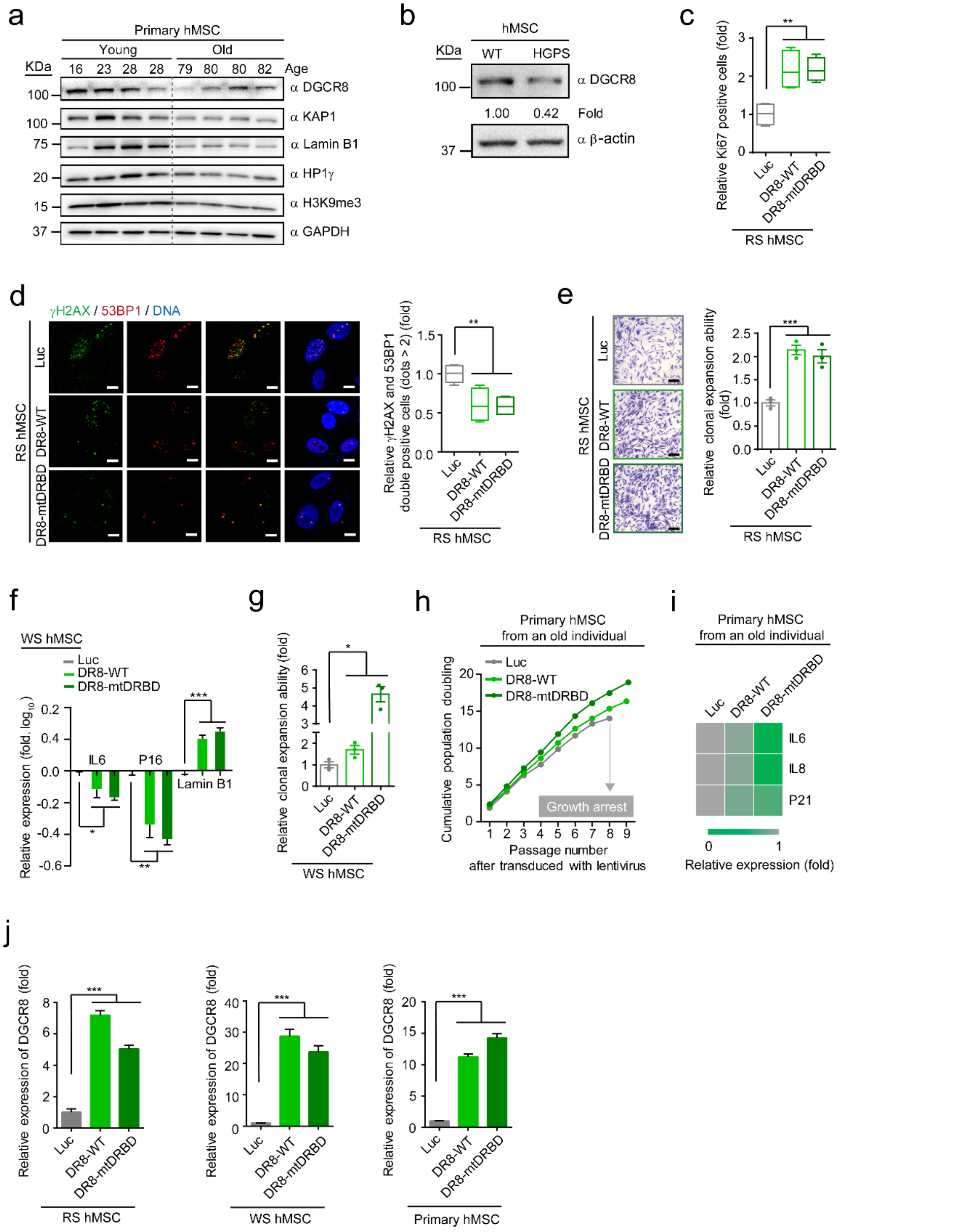

Supplementary Figure 9. DGCR8 plays important roles in regulating hMSC senescence. (**a**) Western blotting analysis of DGCR8, KAP1, Lamin B1, HP1γ, and H3K9me3 in human primary MSCs derived from young and old healthy individuals at passage 7. GAPDH was used as the loading control. (**b**) Western blotting analysis of DGCR8 in HGPS hMSCs. β-actin was used as the loading control. (**c**) Ki67 staining of RS hMSCs transduced with Luc, DR8-WT, or DR8-mtDRBD. Data were presented as mean ± SEM. n=4 images per condition, **$p < 0.01$. Box plots show the median (center line inside the box), upper and lower quartiles (bounds of box), largest and smallest values (whiskers). (**d**) Immunostaining of γH2AX and 53BP1 in RS hMSCs transduced with Luc, DR8-WT, or DR8-mtDRBD. Data were presented as mean ± SEM. n=4 images per condition, **$p < 0.01$. Scale bar, 10 μm. Box plots show the median (center line inside the box), upper and lower quartiles (bounds of box), largest and smallest values (whiskers). (**e**) Clonal formation analysis of RS hMSCs transduced with Luc, DR8-WT, or DR8-mtDRBD. Scale bar, 20 μm. Data were presented as mean ± SEM. n=3 wells per condition, ***$p < 0.001$. (**f**) RT-qPCR detection of IL6, P16, and LMNB1 in WS MSCs transduced with Luc, DR8-WT, or DR8-mtDRBD. Data were presented as mean ± SEM. n=4 wells per condition, *$p < 0.05$, **$p < 0.01$, ***$p < 0.001$. (**g**) Clonal formation analysis of WS hMSCs transduced with Luc, DR8-WT, or DR8-mtDRBD. Data were presented as mean ± SEM. n=3 wells per condition, *$p < 0.05$. (**h**) Growth curve of primary hMSCs from an 80-year-old individual transduced with lentiviruses expressing Luc, DR8-WT, or DR8-mtDRBD. (**i**) Heatmap showing quantitative RT-PCR analysis of the indicated genes in primary hMSCs transduced with lentiviruses expressing Luc, DR8-WT, or DR8-mtDRBD. (**j**) RT-qPCR detection of DGCR8 in RS, WS, or primary hMSCs transduced with lentiviruses expressing Luc, DR8-WT, or DR8-mtDRBD. Data were presented as mean ± SEM. n=3 wells per condition, ***$p < 0.001$. Statistical significances were assessed by a two-tailed unpaired Student's t test.

Supplementary Figure 10

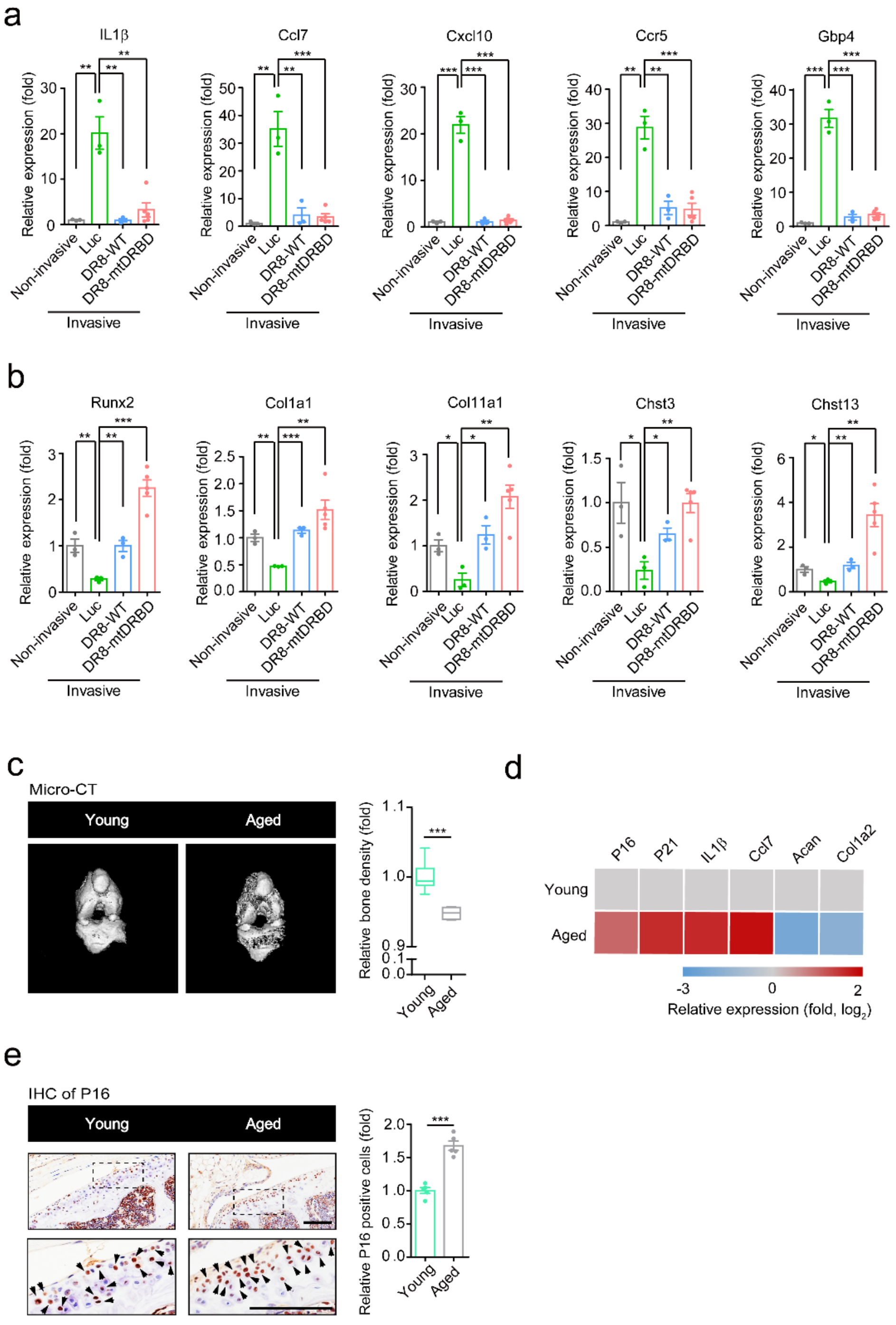

Supplementary Figure 10. The validation of differential expression genes derived from RNA-seq of mouse knee joints. (**a**) Quantitative RT-PCR analysis of downregulated genes in non-invasive (n=3 mice) mouse joints and ACLT joints treated with lentivirus expressing DR8-WT (n=3 mice) or DR8-mtDRBD (n=5 mice), compared with ACLT joints treated with lentivirus expressing Luc (n=3 mice). Data were presented as mean ± SEM. **p < 0.01, ***p < 0.001. (**b**) Quantitative RT-PCR analysis of upregulated genes in non-invasive (n=3 mice) mouse joints and ACLT joints treated with lentivirus expressing DR8-WT (n=3 mice) or DR8-mtDRBD (n=5 mice), compared with ACLT joints treated with lentivirus expressing Luc (n=3 mice). Data were presented as mean ± SEM. *p < 0.05, **p < 0.01, ***p < 0.001. (**c**) Bone density analysis of young and aged mouse joints. Data were presented as mean ± SEM. n=5 mice per group, ***p < 0.001. Box plots show the median (center line inside the box), upper and lower quartiles (bounds of box), largest and smallest values (whiskers). (**d**) Heatmap showing quantitative RT-PCR analysis of the indicated genes in young and aged mouse joints. Expression levels of the indicated genes in aged mouse joints were normalized to that in young mouse joints. (**e**) Left, Immunohistochemistry of P16 expression in young and aged mouse joints. Right, Statistical analysis of P16-positive cells. Scale bar, 100 µm. Data were presented as mean ± SEM. n=5 mice per group, ***p < 0.001. Statistical significances were assessed by a two-tailed unpaired Student's t test.

Supplementary Figure 11

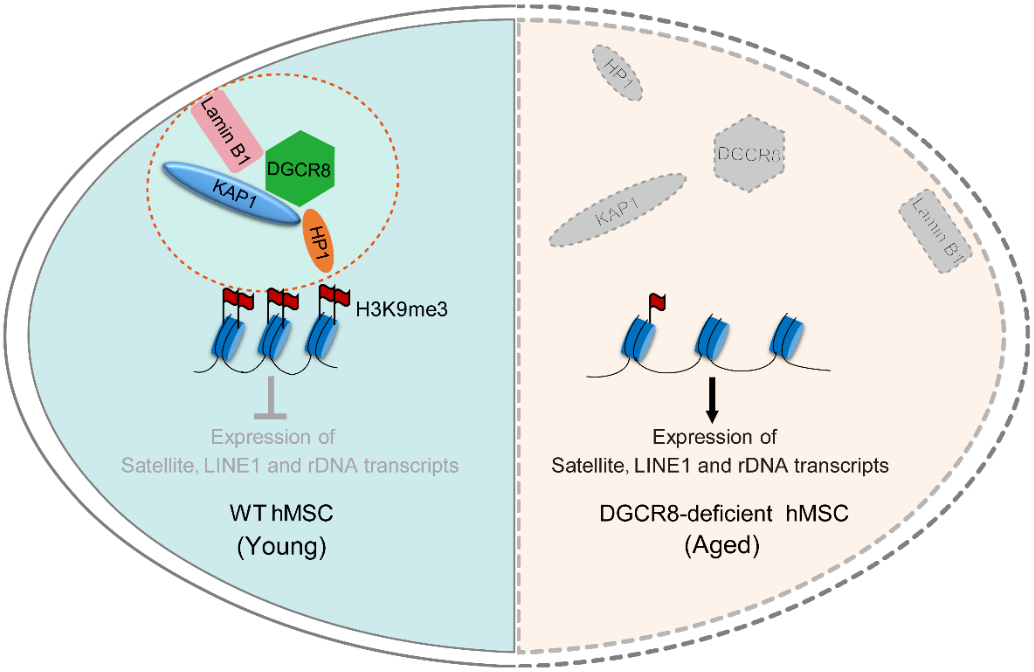

Supplementary Figure 11. A putative model describing the role of DGCR8 in stabilizing heterochromatin. Left, in WT (Young) hMSCs, DGCR8 protein forms a complex with the heterochromatin components KAP1, Lamin B1, and HP1, which together associates with H3K9me3-enriched heterochromatin tethered to nuclear envelope regions. Right, in DGCR8-deficient (Aged) hMSCs, the complex is dissociated due to loss of DGCR8 function, which results in the disorganization of heterochromatin and nuclear lamina, and induces active transcription of heterochromatin-associated sequences.

Supplementary Figure 12

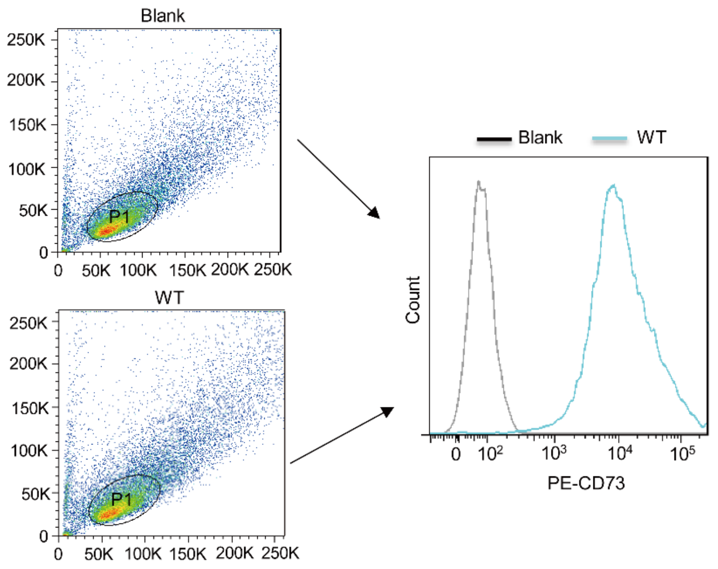

Supplementary Figure 12. A figure exemplifying the gating strategy used in the study. FSC/SSC gates were applied to specify living cells (P1), and then staining positive cells were gated among living cells. The gating panels corresponding to FACS data panels in Figure 2a and Supplementary Figure 2a.

Supplementary Figure 13

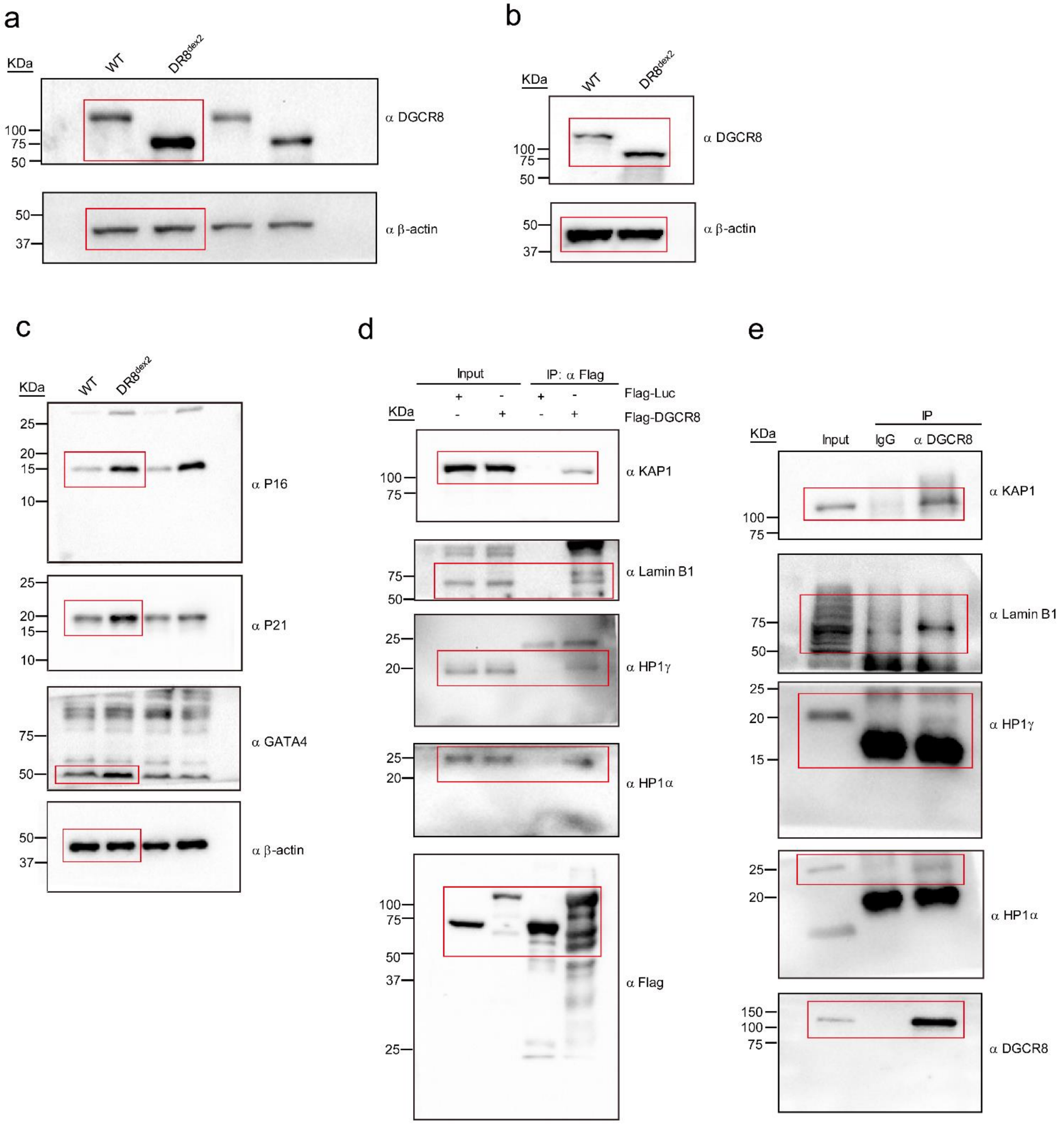

Supplementary Figure 13. Uncropped scans with size marker indications. (**a-e**) Uncropped blots of Figures 1b, 2b, 2f, 4a and 4b. Edited blots were marked by red rectangles.

Supplementary Figure 14

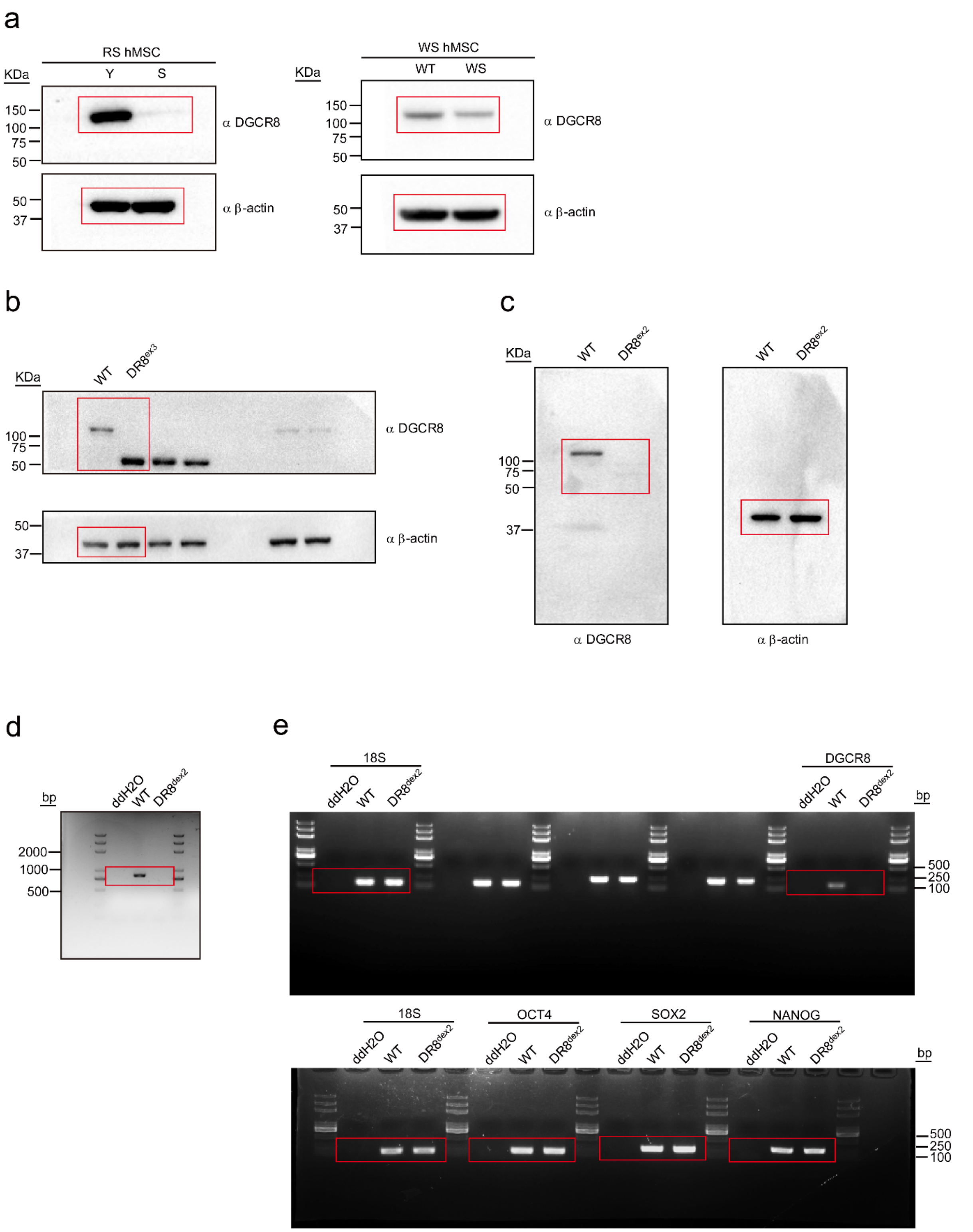

Supplementary Figure 14. Uncropped scans with size marker indications. (**a-e**) Uncropped blots or gels of Figure 6b and Supplementary Figures 1b, 1e, 1l and 1m-1n. Edited blots or gels were marked by red rectangles.

Supplementary Figure 15

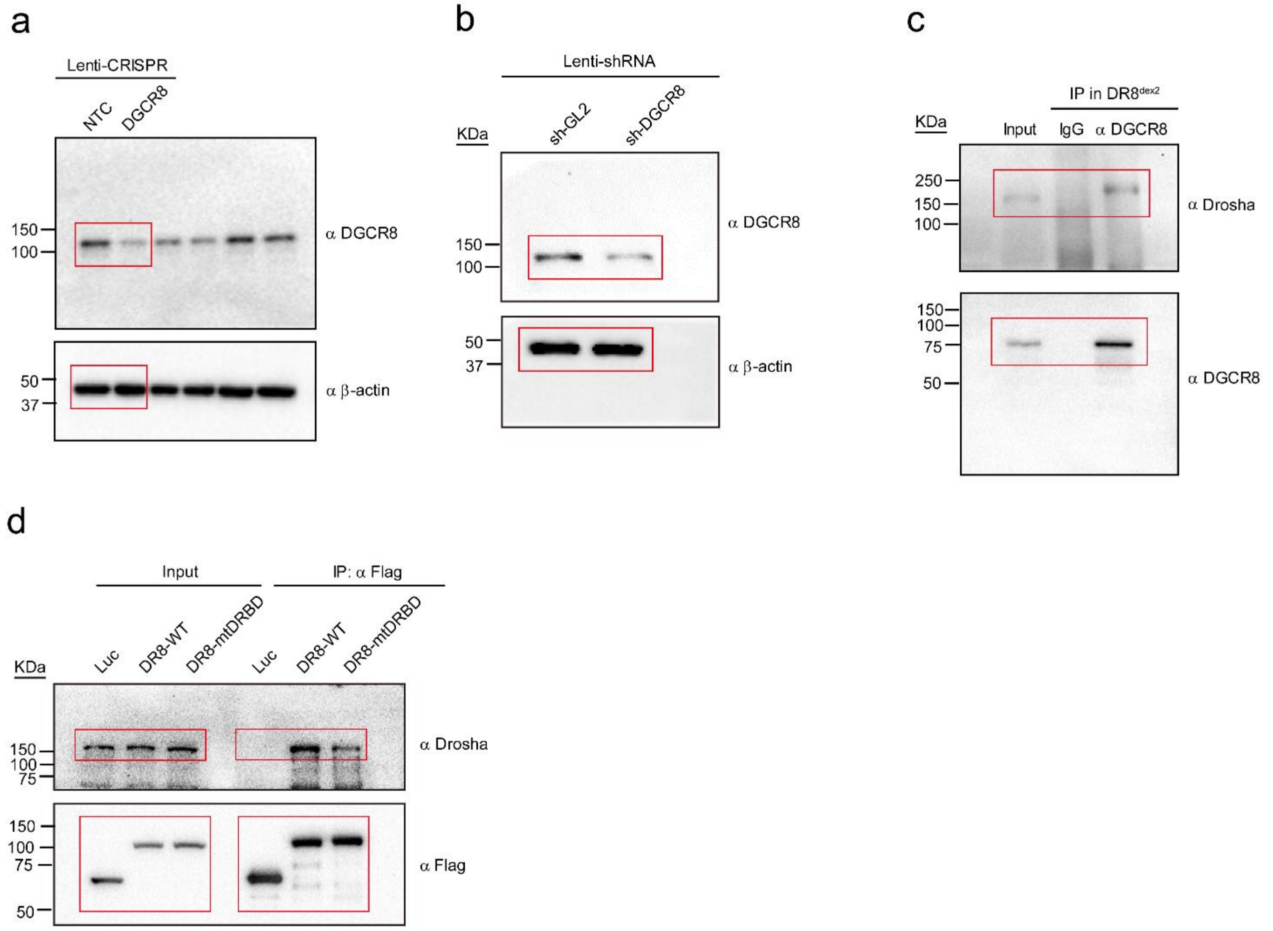

Supplementary Figure 15. Uncropped scans with size marker indications. (**a-d**) Uncropped blots of Supplementary Figures 2g, 2j, 3a and 3e. Edited blots were marked by red rectangles.

Supplementary Figure 16

a

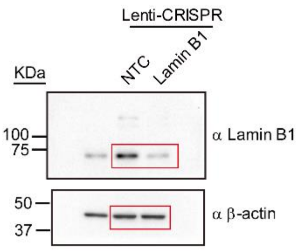

b

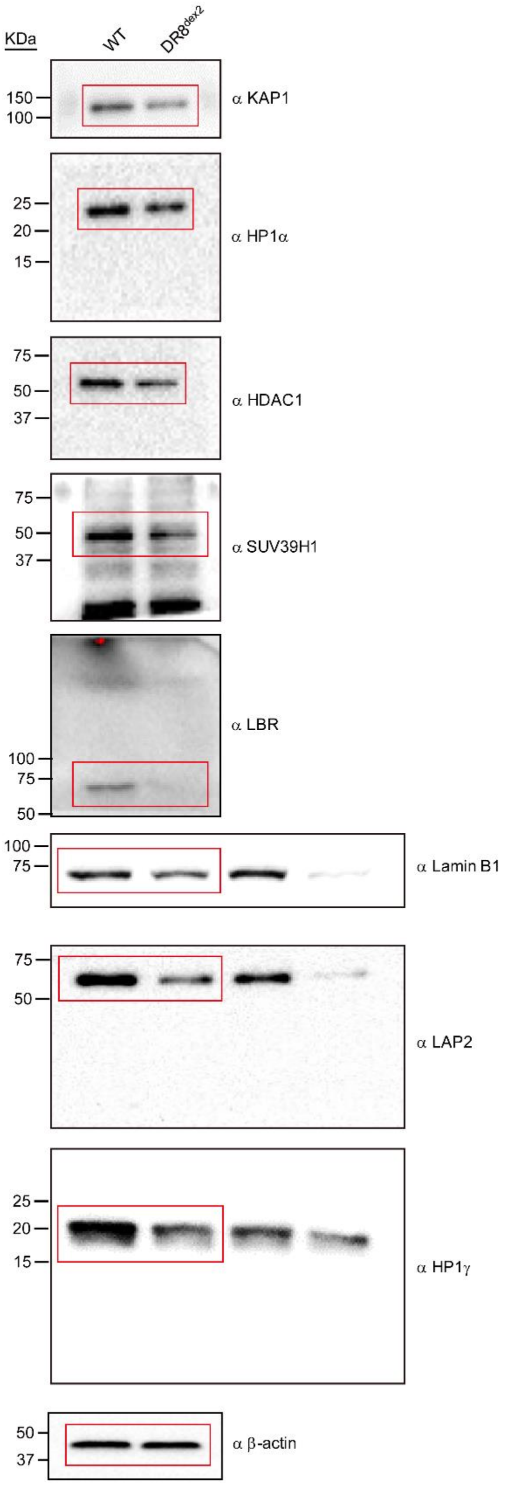

Supplementary Figure 16. Uncropped scans with size marker indications. (**a-b**) Uncropped blots of Supplementary Figures 4c and 4e. Edited blots were marked by red rectangles.

Supplementary Figure 17

a

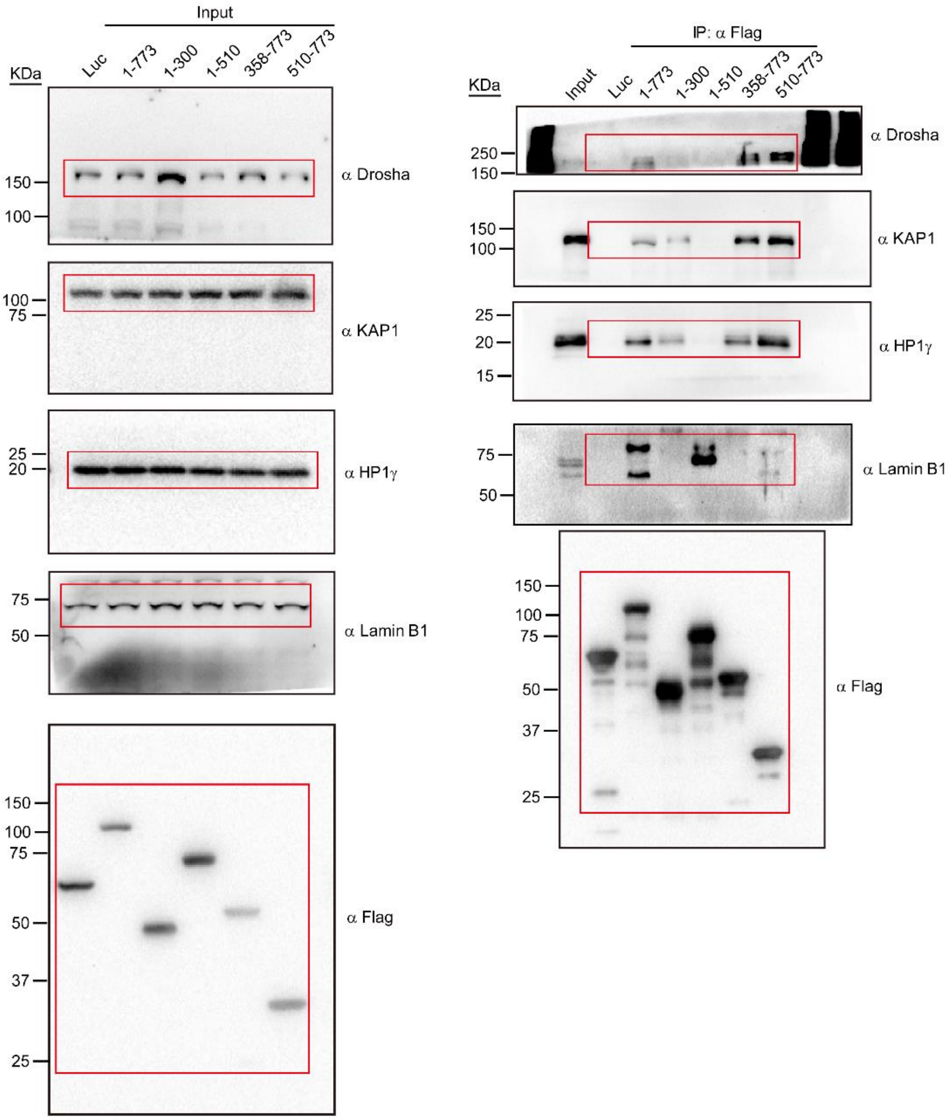

Supplementary Figure 17. Uncropped scans with size marker indications. (**a**) Uncropped blots of Supplementary Figure 7g. Edited blots were marked by red rectangles.

Supplementary Figure 18

a

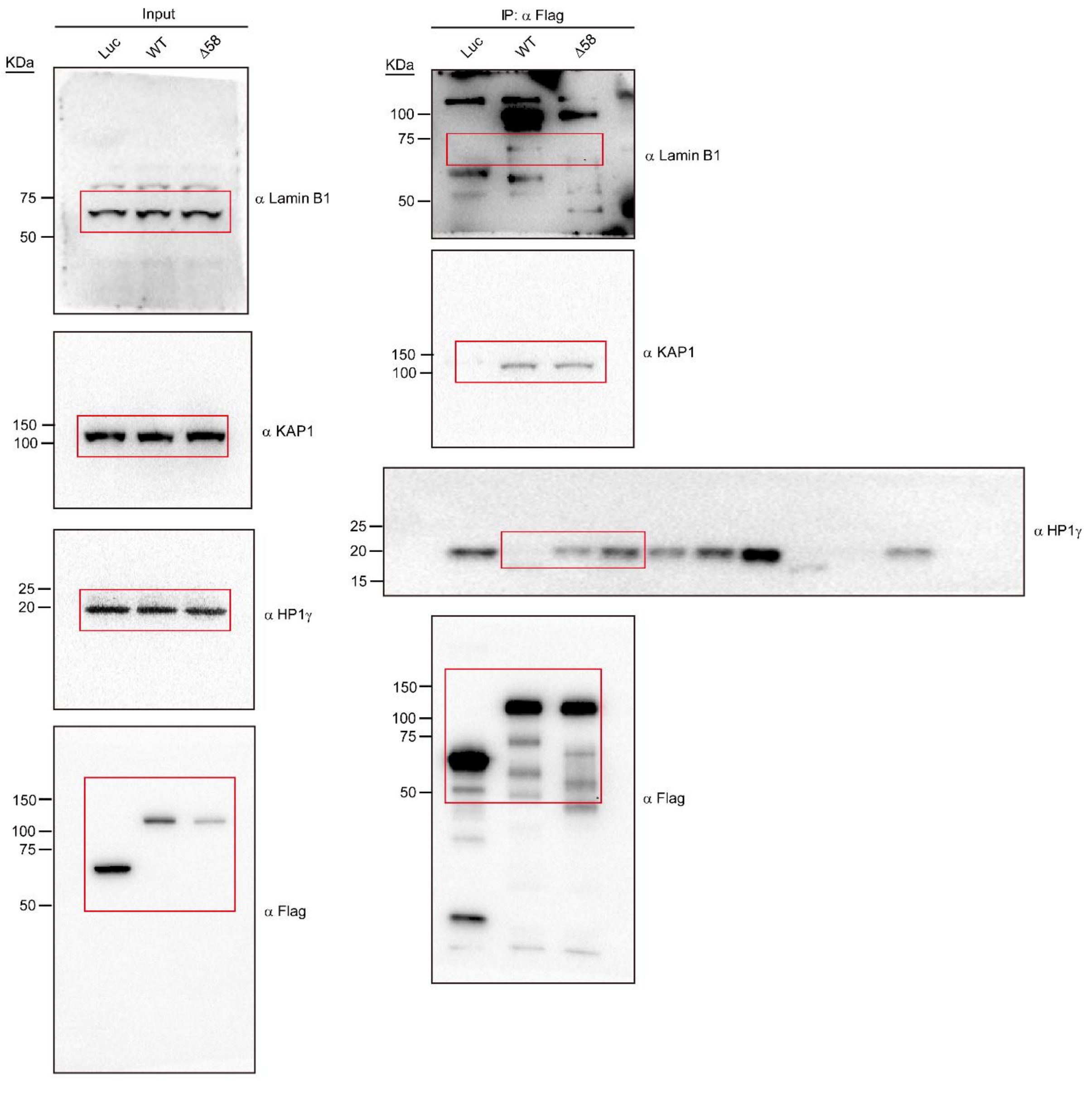

Supplementary Figure 18. Uncropped scans with size marker indications. (**a**) Uncropped blots of Supplementary Figure 7h. Edited blots were marked by red rectangles.

Supplementary Figure 19

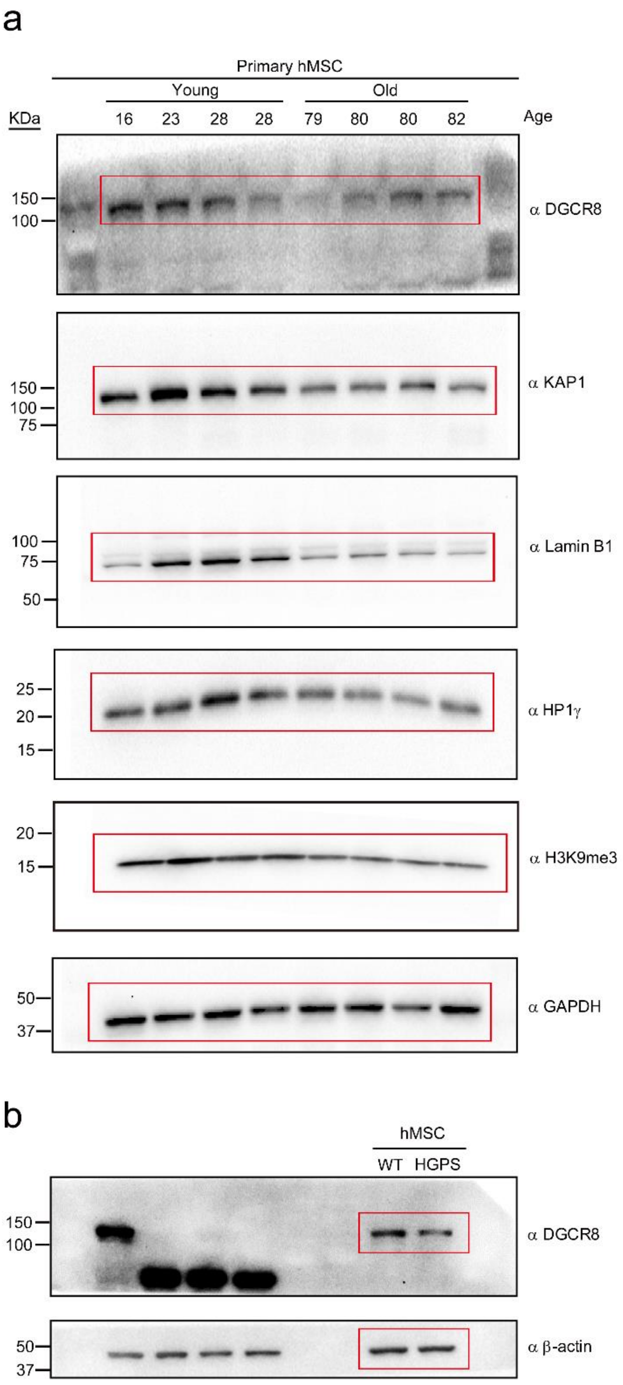

Supplementary Figure 19. Uncropped scans with size marker indications. (**a-b**) Uncropped blots of Supplementary Figures 9a and 9b. Edited blots were marked by red rectangles.
