# Supplementary material for: Stabilizing heterochromatin by DGCR8 alleviates senescence and osteoarthritis
Source: Nat Commun. 2019 Jul 26;10:3329. doi: 10.1038/s41467-019-10831-8 (PMC6659673; doi:10.1038/s41467-019-10831-8)
Supplement: Supplementary file 3 — Description of Additional Supplementary Files [file 41467_2019_10831_MOESM3_ESM.pdf]

Description of Additional Supplementary Files

File Name: Supplementary Data 1

Description: The list and sequences of primers used for qPCR, semi-qPCR, ChIP-qPCR, and PCR.

File Name: Supplementary Data 2

Description: The list and sequences of sgRNAs.

File Name: Supplementary Data 3

Description: The list and sequences of shRNAs.

File Name: Supplementary Data 4

Description: Information on dental pulp-derived primary hMSCs.

File Name: Supplementary Data 5 (corresponding to Supplementary Figure 3c)

Description: The list of differentially expressed miRNAs between WT and $DR8^{dex2}$ hMSCs.

File Name: Supplementary Data 6

Description: The list of interacting proteins of DGCR8 identified by mass spectrometry.

File Name: Supplementary Data 7 (corresponding to Figure 8b)

Description: The list of downregulated and upregulated genes by RNA-seq of non-invasive mouse joints and ACLT joints treated with lentiviruses expressing DR8-WT or DR8-mtDRBD, compared with ACLT joints treated with lentiviruses expressing Luc.
