# Supplementary material for: Stabilizing heterochromatin by DGCR8 alleviates senescence and osteoarthritis
Source: Nat Commun. 2019 Jul 26;10:3329. doi: 10.1038/s41467-019-10831-8 (PMC6659673; doi:10.1038/s41467-019-10831-8)

Figure 1b

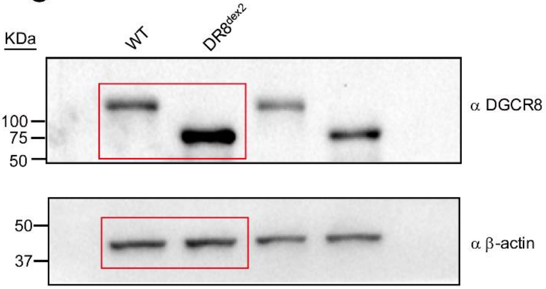

Figure 2b

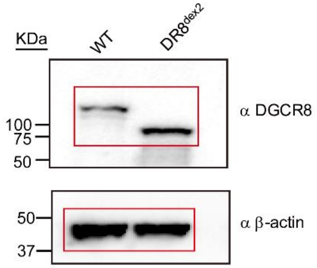

Figure 2f

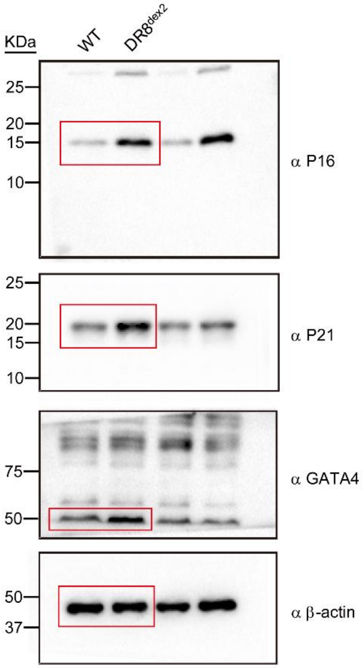

Figure 4a

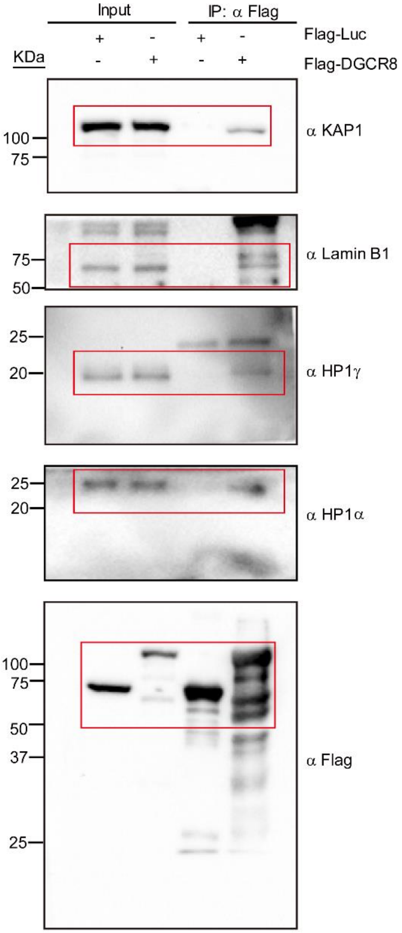

Figure 4b

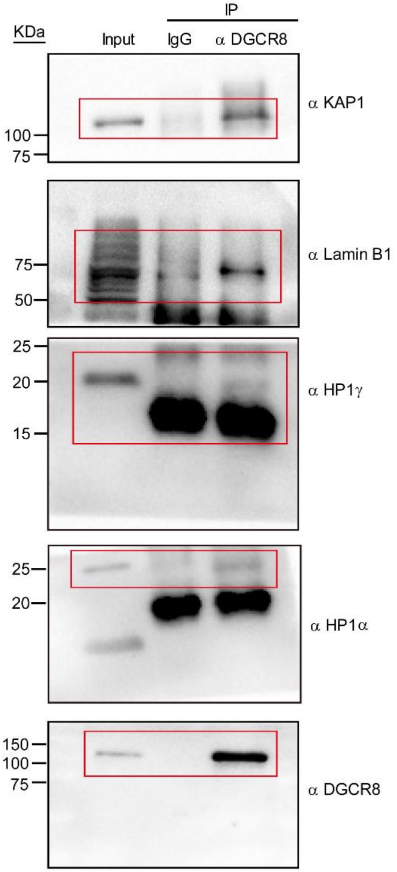

Figure 6b

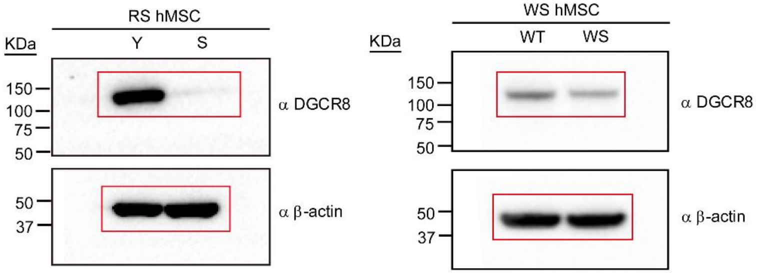

Figure S1b

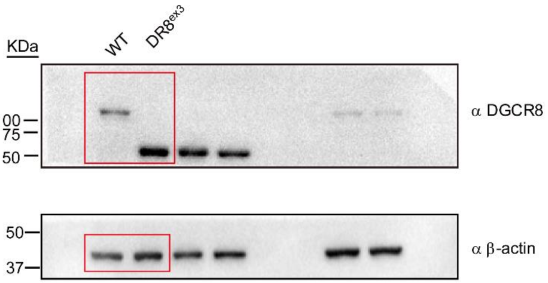

Figure S1e

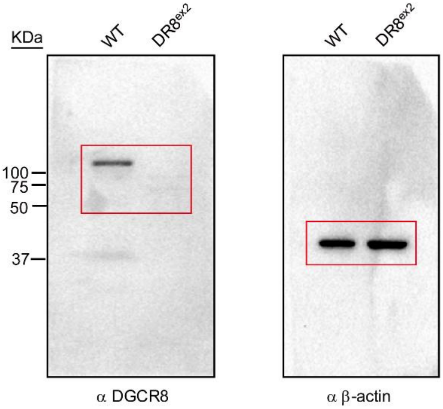

Figure S1l

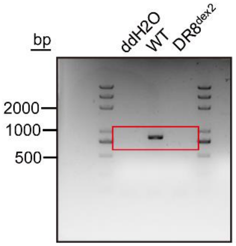

Figure S1m and 1n

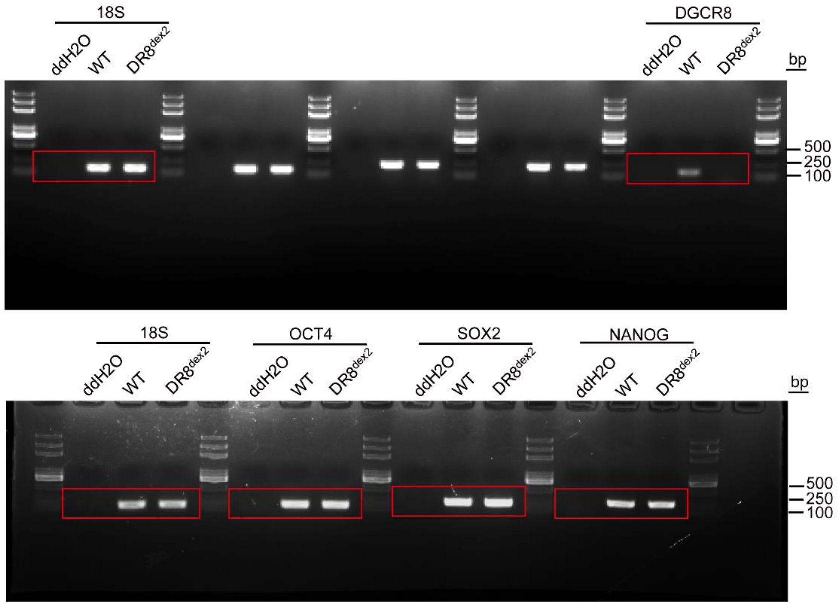

Figure S2g

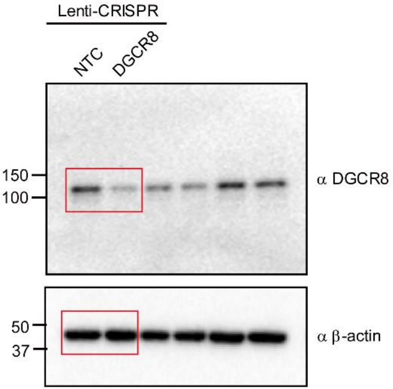

Figure S2j

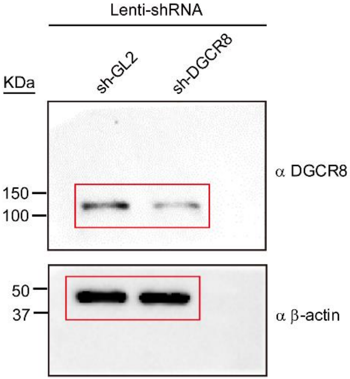

Figure S3a

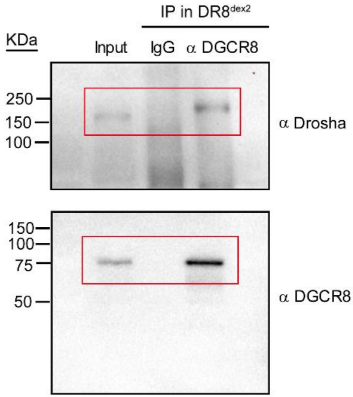

Figure S3e

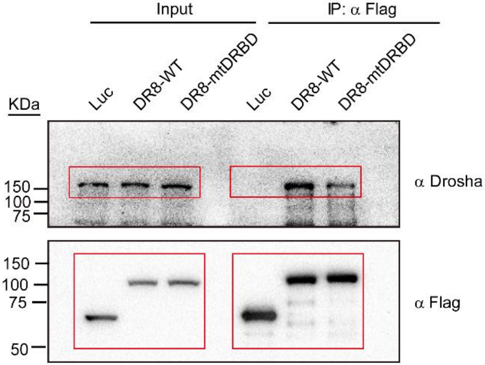

Figure S4c

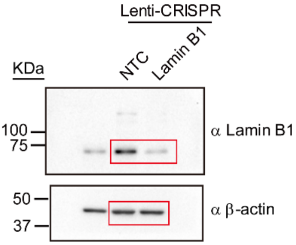

Figure S4e

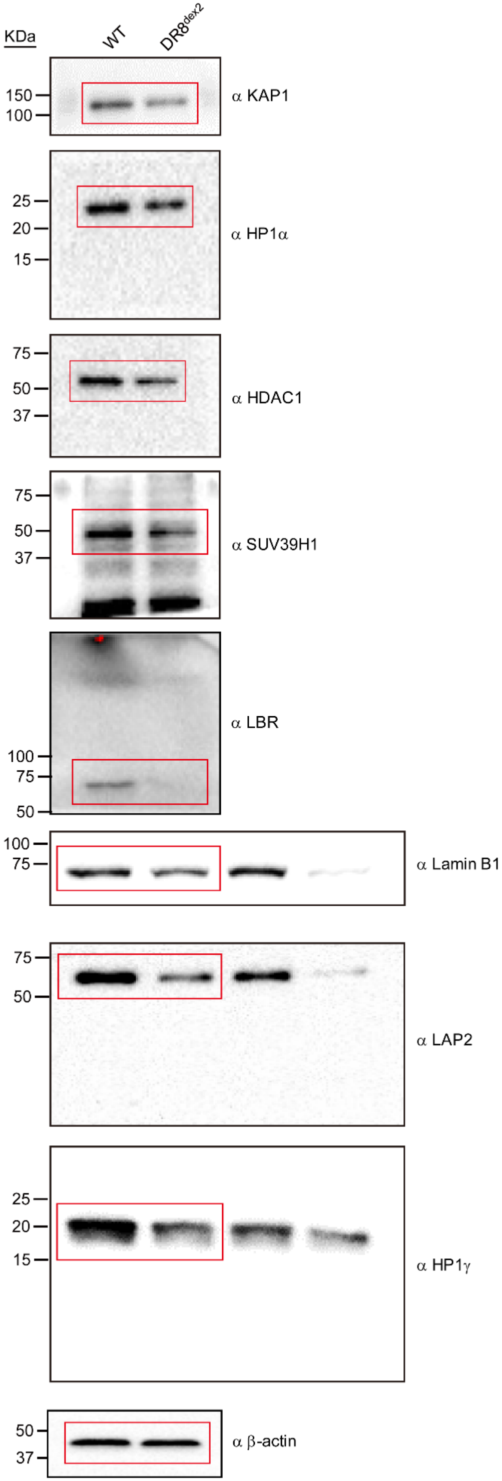

Figure S7g

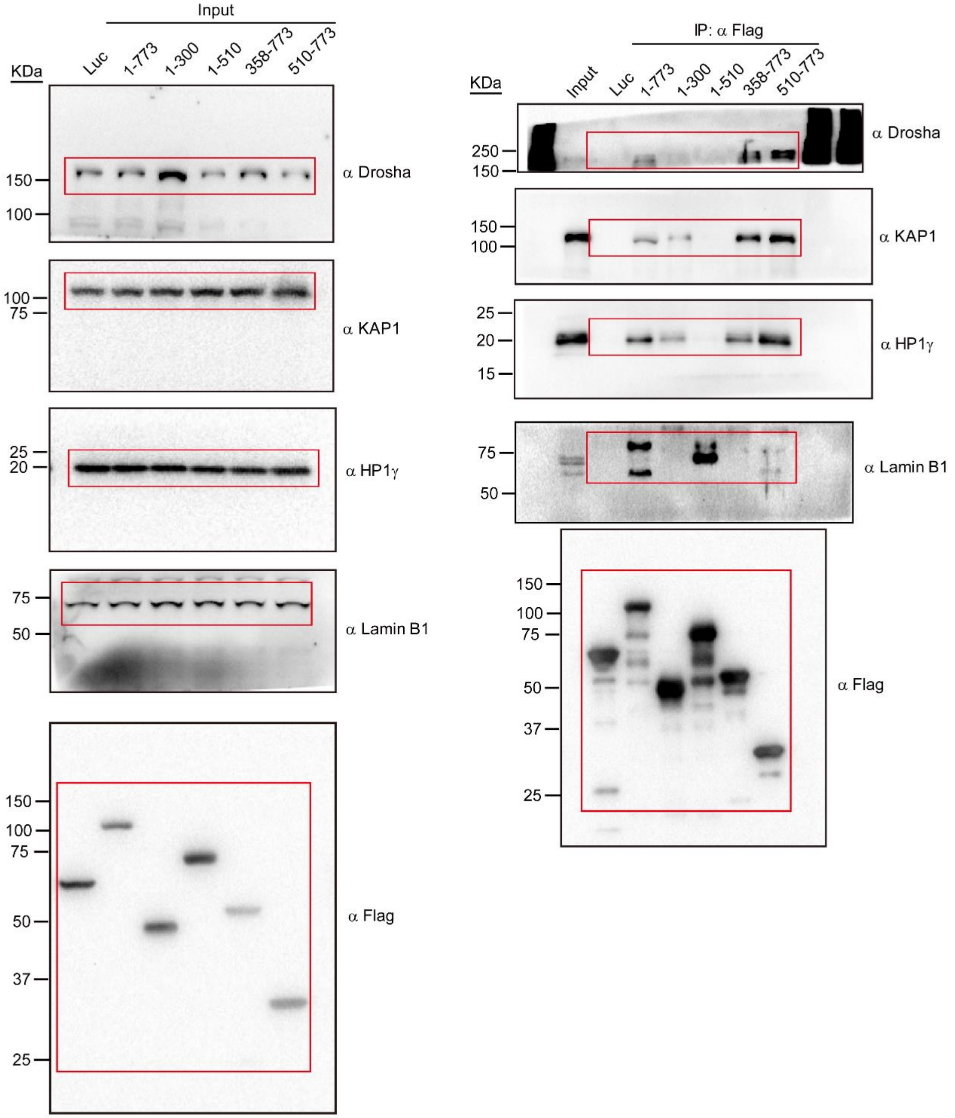

Input
KDa
Luc
1-773
1-300
1-510
358-773
510-773
150
100
α Drosha
100
75
α KAP1
25
20
α HP1γ
75
50
α Lamin B1
150
100
75
50
37
25
α Flag
IP: α Flag
KDa
Input
Luc
1-773
1-300
1-510
358-773
510-773
250
150
α Drosha
150
100
α KAP1
25
20
15
α HP1γ
75
50
α Lamin B1
150
100
75
50
37
25
α Flag

Figure S7h

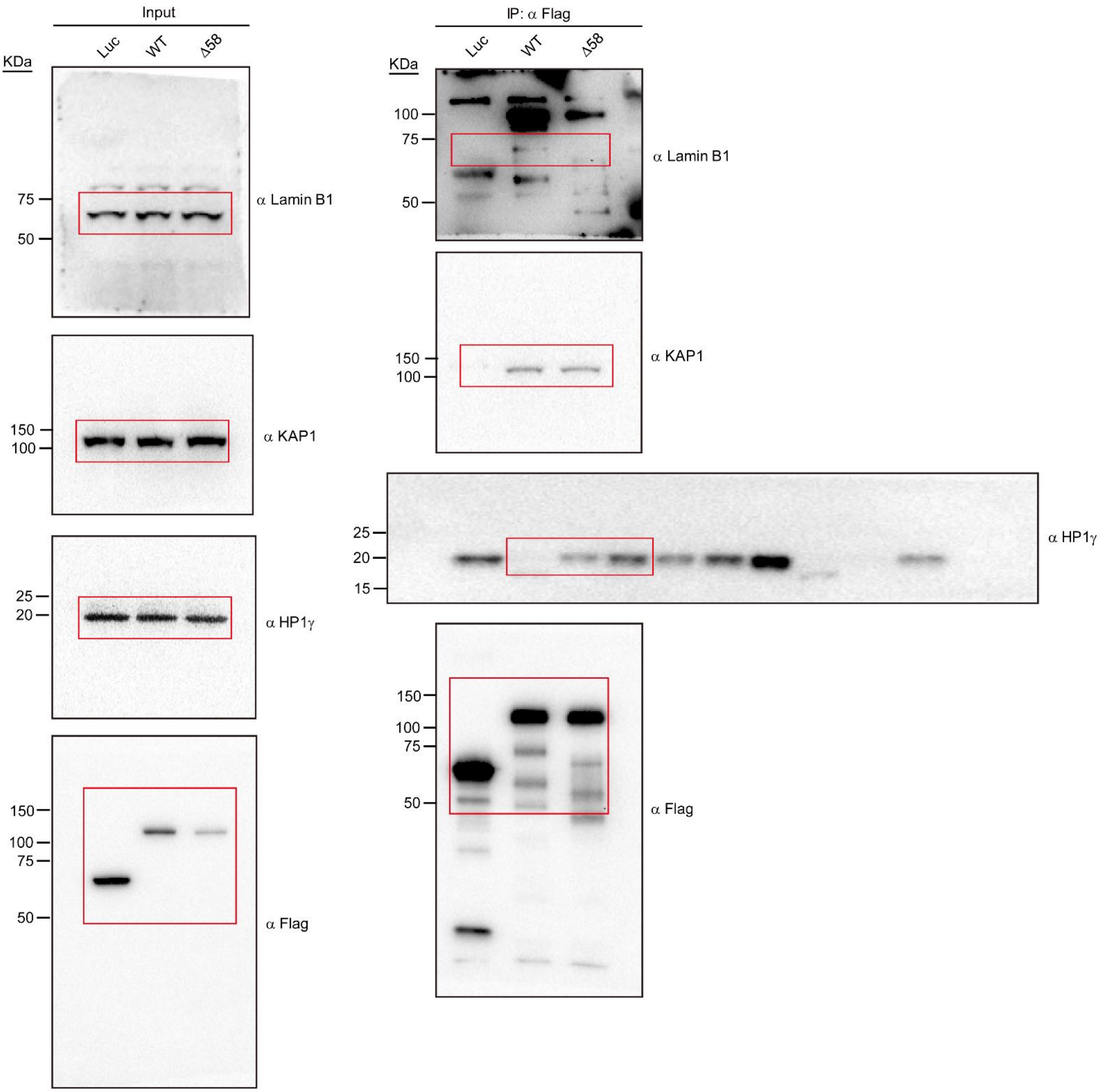
Input
Luc
WT
Δ58
KDa
75
50
α Lamin B1
150
100
α KAP1
25
20
α HP1γ
150
100
75
50
α Flag
IP: α Flag
Luc
WT
Δ58
KDa
100
75
50
α Lamin B1
150
100
α KAP1
25
20
15
α HP1γ
150
100
75
50
α Flag

Figure S9a and 9b

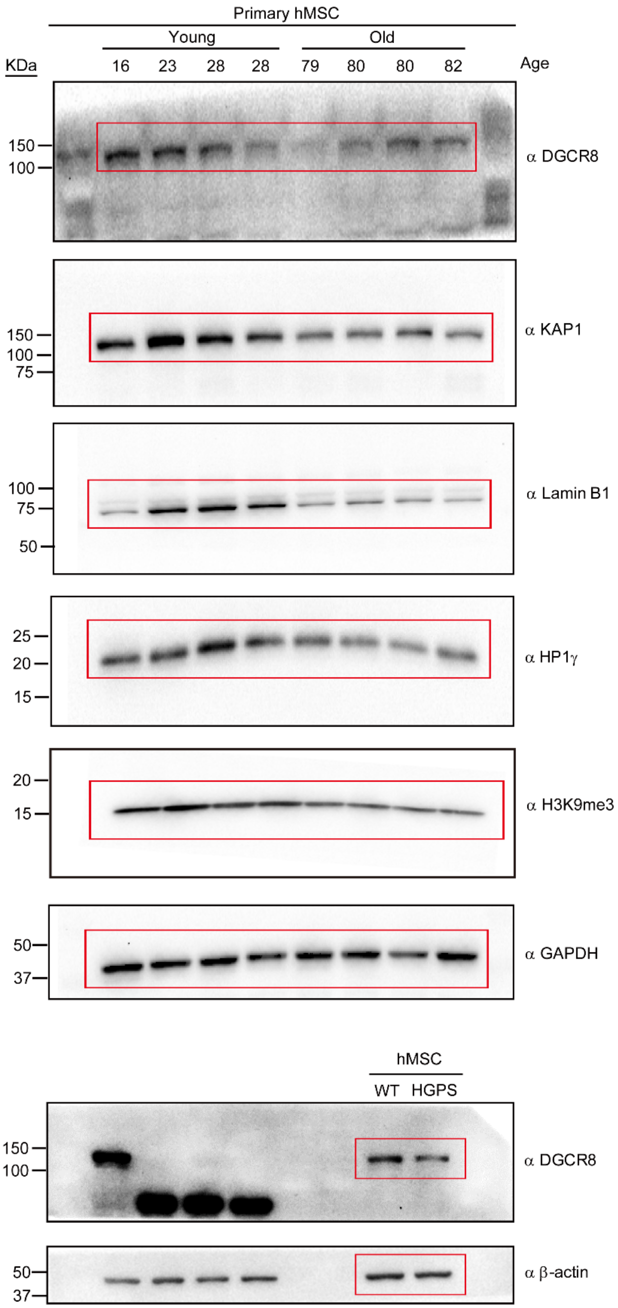

Supplement: Supplementary file 11 — Source Data [file 41467_2019_10831_MOESM11_ESM.zip › Source.pdf]
